# MLX phosphorylation stabilizes the ChREBP-MLX heterotetramer on tandem E-boxes to control carbohydrate and lipid metabolism

**DOI:** 10.1126/sciadv.adt4548

**Published:** 2025-03-12

**Authors:** Carla E. Cadena del Castillo, Onur Deniz, Femke van Geest, Lore Rosseels, Ingrid Stockmans, Marius Robciuc, Sebastien Carpentier, Bettina K. Wölnerhanssen, Anne Christin Meyer-Gerspach, Ralph Peterli, Ville Hietakangas, Mitsugu Shimobayashi

**Affiliations:** ^1^ Clinical and Experimental Endocrinology, Department of Chronic Diseases and Metabolism, KU Leuven, Leuven, Belgium.; ^2^ Faculty of Biological and Environmental Sciences, University of Helsinki, Helsinki, Finland.; ^3^ Institute of Biotechnology, Helsinki Institute of Life Science, University of Helsinki, Helsinki, Finland.; ^4^ Facility for Systems Biology Based Mass Spectrometry, KU Leuven, Leuven, Belgium.; ^5^ St. Clara Research Ltd, St. Claraspital, Basel, Switzerland.; ^6^ University of Basel, Basel, Switzerland.; ^7^ Clarunis, University Digestive Health Care Center, St. Clara Hospital and University Hospital Basel, Basel, Switzerland.

## Abstract

Carbohydrate-responsive element binding protein (ChREBP) and Max-like protein X (MLX) form a heterodimeric transcription factor complex that couples intracellular sugar levels to carbohydrate and lipid metabolism. To promote the expression of target genes, two ChREBP-MLX heterodimers form a heterotetramer to bind a tandem element with two adjacent E-boxes, called carbohydrate-responsive element (ChoRE). How the ChREBP-MLX hetero-tetramerization is achieved and regulated remains poorly understood. Here, we show that MLX phosphorylation on an evolutionarily conserved motif is necessary for the heterotetramer formation on the ChoRE and the transcriptional activity of the ChREBP-MLX complex. We identified casein kinase 2 (CK2) and glycogen synthase kinase 3 (GSK3) as MLX kinases. High intracellular glucose-6-phosphate accumulation inhibits MLX phosphorylation and heterotetramer formation on the ChoRE, impairing ChREBP-MLX activity. Physiologically, MLX phosphorylation is necessary in *Drosophila* to maintain sugar tolerance and lipid homeostasis. Our findings suggest that MLX phosphorylation is a key mechanism for the ChREBP-MLX heterotetramer formation to regulate carbohydrate and lipid metabolism.

## Teaser

CK2-mediated MLX phosphorylation is an evolutionarily conserved signaling pathway that controls sugar and lipid metabolism.

## INTRODUCTION

Transcriptional regulation plays a central role for organisms to adapt their metabolism in response to nutrient availability. The basic helix-loop-helix/leucine zipper (bHLH/LZ) Mondo family transcription factors (TFs) promote the expression of carbohydrate-induced genes. In mammals, there are two Mondo paralogs, carbohydrate-responsive element binding protein (ChREBP) and MondoA (*1*, *2*). ChREBP controls the expression of genes encoding glycolytic and lipogenic enzymes in the liver and adipose tissue to promote energy storage (*3*). Previous studies suggested that the glucose metabolite glucose-6-phosphate (G6P) promotes ChREBP transcriptional activity (*4*–*6*), although the underlying molecular mechanism is still unclear.

To promote the expression of its target genes, ChREBP forms a complex with Max-like protein X (MLX) (*7*–*11*). ChREBP-MLX complex specifically binds to two tandem E-box motifs separated by five nucleotides, defined as the carbohydrate response element (ChoRE) (*12*). Notably, endogenous E-boxes in the ChoRE sequence do not perfectly match the canonical tandem E-box sequence (CACGTGxxxxxCACGTG) (*13*), resulting in a weak recognition by the ChREBP-MLX complex. To overcome this weak recognition and promote the expression of the target genes, two ChREBP-MLX heterodimers need to coordinately bind to the naturally occurring imperfect E-boxes and form a heterotetramer (*9*). It was demonstrated that the loop region of the HLH domain of MLX is critical for the ChREBP-MLX heterotetramer formation on the ChoRE (*9*). However, the regulation and physiological role of the tetramer formation remain poorly understood. Here, we uncovered an evolutionarily conserved signaling pathway that controls the ChREBP-MLX heterotetramer formation on the ChoRE, ChREBP-MLX activity, and thereby sugar response.

## RESULTS

### MLX phosphorylation is necessary for ChREBP-MLX transcriptional activity

**Fig. 1.**
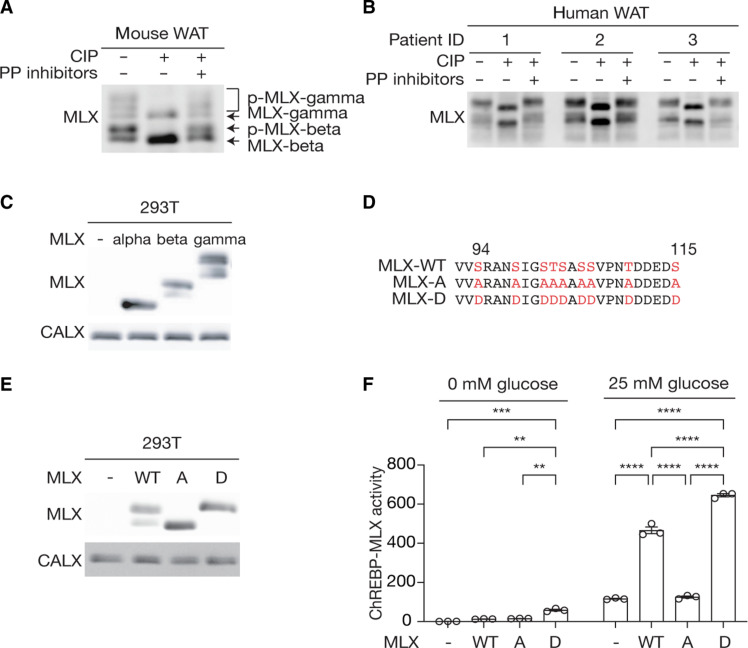
MLX phosphorylation promotes ChREBP-MLX activity. (**A**) and (**B**) MLX phosphorylation in mouse (A) and human (B) white adipose tissue (WAT) lysates treated with calf intestine phosphatase (CIP) or CIP and phosphatase (PP) inhibitors. *n* = 3. (**C**) MLX phosphorylation in 293T cells expressing the MLX isoforms alpha, beta, or gamma. CALX serves as a loading control. *N* = 3. (**D**) Amino acid sequence alignment of wild-type MLX (MLX-WT), phospho-deficient MLX-A, and phospho-mimetic MLX-D. (**E**) MLX phosphorylation in 293T cells expressing MLX-WT, MLX-A, or MLX-D. *N* = 3. (**F**) ChREBP-MLX luciferase reporter activity in 293T cells expressing ChREBP, HK2 and MLX-WT, MLX-A, or MLX-D. Cells were starved for glucose overnight and treated with 25 mM glucose for 3 hours. Two-way ANOVA, **P < 0.01, ***P < 0.001, and ****P < 0.0001. *N* = 3.

Since protein phosphorylation frequently regulates TF activity (*14*), we explored whether phosphorylation of MLX regulates the ChREBP-MLX function. MLX appeared as multiple bands in immunoblots of protein lysates from mouse and human white adipose tissue (WAT), with slow-migrating bands sensitive to phosphatase treatment (Fig. 1, A and B). The faster-migrating bands in the phosphatase-treated lysates correspond to the MLX isoforms, MLX-beta and MLX-gamma (Fig. 1, A and B) (*15*). These data suggest that MLX is phosphorylated in mammalian WAT. To investigate the role and regulation of MLX phosphorylation, we identified the phosphorylation sites on MLX. Phosphoproteome studies have identified 22 putative serine (S), threonine (T), and tyrosine (Y) phosphorylation sites in four regions on MLX (fig. S1A) (*16*). To determine the phosphorylation sites responsible for the band shift, we analyzed MLX phosphorylation in 293T cells transiently expressing MLX-alpha, MLX-beta, or MLX-gamma. MLX-beta and MLX-gamma, but not MLX-alpha, displayed slower migrating bands (Fig. 1C), suggesting that the MLX phosphorylation detected by the band shift is specific to the regions common to MLX-beta and MLX-gamma (P80 to D111). The mutation of S94, S98, S101, T102, S103, S105, S106, T110, and S115 to alanine (MLX-A) or aspartic acid (MLX-D) (Fig. 1D) prevented and mimicked MLX phosphorylation, respectively (Fig. 1E and fig. S1B). These data show that mammalian MLX is phosphorylated on the S/T residues between S94 and S115.

To study the role of MLX phosphorylation, we examined ChREBP-MLX transcriptional activity by a reporter system (*3*–*5*, *9*, *17*) (fig. S1C). We optimized the reporter system by coexpressing hexokinase 2 (HK2) to robustly monitor ChREBP-MLX activity (*4*, *5*, *18*) (fig. S1, D and E). Compared with MLX-WT, MLX-A and MLX-D displayed reduced and increased transcriptional activity, respectively (Fig. 1F). These data suggest that MLX phosphorylation is necessary for the ChREBP-MLX activity.

ChREBP is dephosphorylated and activated in response to high glucose (*19*). To investigate the importance of MLX phosphorylation compared with ChREBP dephosphorylation, we examined the ChREBP-MLX transcriptional activity with a phospho-deficient ChREBP (ChREBP-A:S140A, S196A, S626A, and T665A). MLX-A inhibited the transcriptional activity with ChREBP-WT and ChREBP-A (fig. S1F), suggesting that MLX dephosphorylation overrides ChREBP dephosphorylation to regulate ChREBP-MLX activity.

### MLX phosphorylation is required for the sugar response in *Drosophila*

**Fig. 2.**
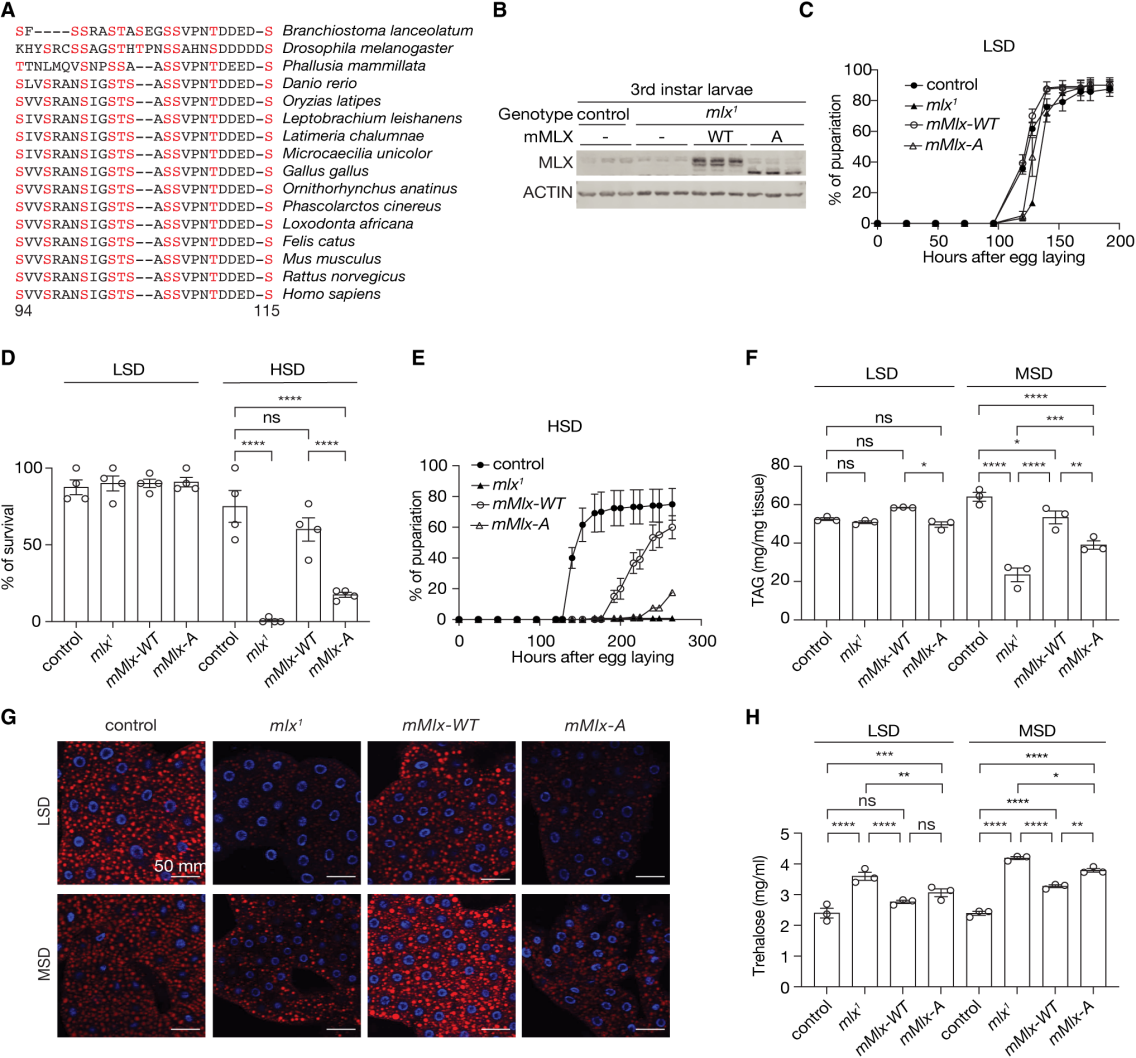
MLX phosphorylation on an evolutionarily conserved motif promotes the sugar response in *Drosophila*. (**A**) Amino acid sequence alignment of MLX phosphorylation sites across different animal species. (**B**) MLX phosphorylation in third instar control or *mlx* null (*mlx^1^*) larvae complemented with fat body–specific expression of mouse MLX-WT or MLX-A (*mMlx-WT* or *mMlx-A*, respectively). ACTIN serves as a loading control. *n* = 3. (**C**) Pupariation of control, *mlx^1^*, *mMlx-WT* or *mMlx-A* larvae grown in low-sugar diet (LSD; 10% yeast). *N* = 4. (**D**) Survival of control, *mlx^1^*, *mMlx-WT*, or *mMlx-A* larvae grown on LSD or high-sugar diet (HSD; 10% yeast and 15% sucrose). Two-way ANOVA, ****P < 0.0001; ns, not significant. *N* = 4. (**E**) Pupariation of control, *mlx^1^*, *mMlx-WT*, or *mMlx-A* larvae grown on HSD. *N* = 4. (**F**) Triacylglycerol (TAG) levels of third instar control, *mlx^1^*, *mMlx-WT*, or *mMlx-A* larvae grown on LSD or medium-sugar diet (MSD; 10% yeast and 5% sucrose). Two-way ANOVA, *P < 0.05, **P < 0.01, ***P < 0.001, and ****P < 0.0001; ns, not significant. *N* = 3. (**G**) Neutral lipid staining of the fat body in third instar control, *mlx^1^*, *mMlx-WT*, or *mMlx-A* larvae grown in LSD or MSD. *n* = 5. (**H**) Hemolymph trehalose levels in third instar control, *mlx^1^*, *mMlx-WT*, or *mMlx-A* larvae in LSD or MSD. Two-way ANOVA, *P < 0.05, **P < 0.01, ***P < 0.001, and ****P < 0.0001; ns, not significant. *N* = 3.

MLX protein and phosphorylation sites are highly conserved across animalia (Fig. 2A and fig. S2, A and B). In *Drosophila*, the *mlx* null mutants (*mlx^1^*) displayed larval sugar intolerance, reduced lipid storage, and elevated circulating sugar levels (*10*). To demonstrate the evolutionarily conserved role and the physiological relevance of MLX phosphorylation, we complemented loss of *mlx* with transgenes encoding mouse MLX-WT or MLX-A specifically in the fat body, the fly functional counterpart of the liver and adipose tissue (*Fb>mMlx-WT, mlx^1^* and *Fb>mMlx-A, mlx^1^*). These transgenic flies are hereafter referred to as *mMlx-WT* and *mMlx-A*. In larval extracts, mMLX-A displayed faster electrophoretic mobility compared to mMLX-WT, consistent with phosphorylation of the conserved serine/threonine residues between S94 and S115 (Fig. 2B). As shown before (*10*), *mlx^1^* larvae displayed nearly normal development on a low-sugar diet (LSD; 10% yeast), which was not affected by the expression of *mMlx-WT* or *mMlx-A* (Fig. 2, C and D). The *mlx^1^* larvae neither grew nor pupariate on a high-sugar diet (HSD; 10% yeast + 15% sucrose) (*10*), but this sugar intolerance was partially rescued in *mMlx-WT* larvae (Fig. 2, D and E). The rescue effect observed in *mMlx-A* larvae was substantially weaker compared to *mMlx-WT* larvae (Fig. 2, D and E), consistent with the functional importance of MLX phosphorylation in supporting larval development and survival on a HSD.

Together with the ChREBP ortholog Mondo, *Drosophila* Mlx has a conserved role in promoting lipogenesis in response to sugar feeding (*20*). Therefore, we next examined triacylglycerol (TAG) levels in third instar larvae grown on either LSD or a medium-sugar diet (MSD; 10% yeast + 5% sucrose), which, unlike HSD, allows larval development of *mlx^1^* mutants. In larvae grown on LSD, we observed a slight reduction in whole-body TAG levels in *mMlx-A* larvae, compared to *mMlx-WT* (Fig. 2F). Compared to controls, *mlx^1^* larvae grown on MSD displayed reduced TAG levels, which were rescued by fat body–specific expression of mMLX-WT (Fig. 2F). Notably, the rescue of TAG levels was substantially compromised by fat body–specific expression of mMLX-A (Fig. 2F). To analyze fat body lipid storage, we stained neutral lipids of third instar larvae with LipidTOX (Fig. 2G). Compared with control and *mMlx-WT* larvae, both *mlx^1^* and *mMlx-A* larvae displayed reduced lipid droplet volumes in both LSD and MSD conditions (Fig. 2G and fig. S2C). The fat body cells in *mlx^1^* and *mMLX-A* larvae were smaller than those in control and *mMlx-WT* (Fig. 2G). Reduced lipid volumes were observed in *mlx^1^* and *mMlx-A* larvae, even after normalization to cell size (fig. S2D). These data suggest that MLX phosphorylation is required for sugar-mediated lipid storage in *Drosophila*.

Impaired Mondo/ChREBP-MLX functions in the fat body in flies and adipose tissue in mice cause high circulating sugar levels (*10*, *18*, *20*, *21*). Insect hemolymph contains low levels of diet-derived free glucose and much higher levels of the nonreducing disaccharide trehalose, which is synthesized by the fat body (*22*, *23*). Thus, we examined trehalose and glucose levels in the hemolymph of larvae fed with LSD or MSD. Compared to control larvae, hemolymph trehalose levels were elevated in *mlx^1^* larvae in both LSD- and MSD-fed conditions (Fig. 2H). *mMlx-WT* expression partially rescued the hemolymph trehalose levels, while this rescue effect was substantially blunted in the phospho-deficient mutant line in MSD-fed conditions. Hemolymph glucose levels were not significantly different in LSD-fed conditions, likely due to low abundance and high variability (fig. S2E). However, *mlx^1^* and *mMlx-A* larvae had significantly higher hemolymph glucose levels, compared to control larvae in MSD-fed conditions (fig. S2E). Overall, the data from *Drosophila* indicate that MLX phosphorylation has a physiological role in maintaining organismal sugar tolerance and controlling lipid and carbohydrate homeostasis.

### MLX phosphorylation stabilizes the heterotetrametric ChREBP-MLX complex on ChoRE

**Fig. 3.**
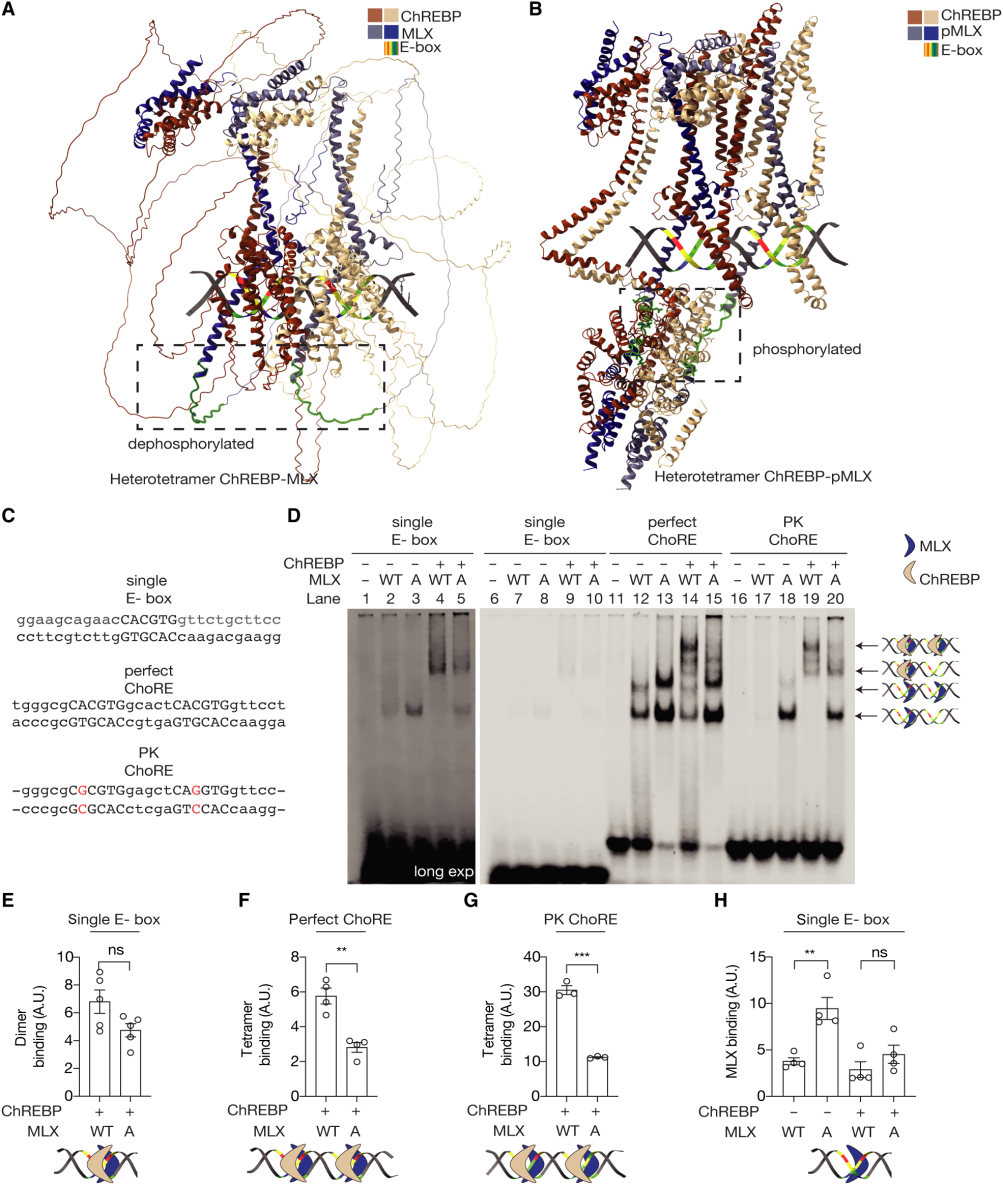
MLX phosphorylation is required for the binding of ChREBP-MLX heterotetrametric complex to the ChoRE. (**A**) and (**B**) A model of ChREBP-MLX (A) or ChREBP-pMLX (B) structure on the PK ChoRE by AlphaFold 3. (**C**) Sequences of single E-box, perfect ChoRE, or PK ChoRE probes. (**D**) Binding of MLX and/or ChREBP to single E-box, perfect ChoRE, or PK ChoRE probes. (**E** to **H**) Quantification in (D). One-way ANOVA or *t* test, **P < 0.01 and ***P < 0.001; ns, not significant. *N* = 3 to 4.

Having established the physiologically important role of MLX phosphorylation in the transcriptional activity and the sugar response (Figs. 1 and 2), we investigated the role of MLX phosphorylation. Protein phosphorylation has been linked to stability of other bHLH/LZ TFs such as Myc (*24*). However, protein stability was not affected by MLX phosphorylation (fig. S3A). We next investigated whether MLX phosphorylation controls the ChREBP-MLX activity via ChoRE binding. First, we modeled two ChREBP-MLX complexes with the ChoRE sequence from the promoter region of the ChREBP-MLX target gene encoding pyruvate kinase by AlphaFold 3 (*25*). The model showed that the MLX phosphorylation motif lies in a flexible region close to the alpha-helix which binds to the E-box (Fig. 3A). A model with phosphorylated MLX showed a drastic conformational change in ChREBP-MLX dimers, stabilizing the flexible region as alpha-helices (Fig. 3B). Furthermore, the model predicted that MLX phosphorylation promotes interdimer interactions between the two ChREBP-MLX dimers, although the confidence score for this interdimer region is very low (fig. S3B). These models suggest that MLX phosphorylation may stabilize the ChREBP-MLX tetramer on the ChoRE sequence.

To experimentally validate the model, we examined the binding affinity of purified ChREBP and MLX for a single perfect E-box, a perfect ChoRE (two tandem perfect E-boxes), and the PK ChoRE sequence by electrophoretic mobility shift assay (EMSA) (Fig. 3, C and D; and fig. S3, C and D). The binding of ChREBP-MLX and ChREBP-MLX-A heterodimer to the single E-box, perfect ChoRE, and PK ChoRE was similar [Fig. 3, D (lanes 4 versus 5, 14 versus 15, and 19 versus 20) and E; and fig. S3, E and F], suggesting that MLX phosphorylation is not required for the MLX-ChREBP dimer formation or recognition of the E-box by a ChREBP-MLX heterodimer. Intriguingly, the binding of ChREBP-MLX heterotetramer to the perfect ChoRE and PK ChoRE was strongly impaired in the absence of MLX phosphorylation [Fig. 3, D (lanes 14 versus 15 and 19 versus 20), F, and G]. These data validate the AlphaFold 3 model and suggest that MLX phosphorylation is required for the stability of the ChREBP-MLX heterotetramer binding to the ChoRE.

Notably, MLX-A displayed greater ChREBP-independent affinity for the single E-box, perfect ChoRE, and PK ChoRE than MLX-WT [Fig. 3D (lanes 2 versus 3, 12 versus 13, and 17 versus 18) and H; and fig. S3, G and H]. It remains to be determined how the ChREBP-independent MLX E-box binding is achieved.

### CK2 and GSK3 sequentially phosphorylate MLX to promote ChREBP-MLX activity

**Fig. 4.**
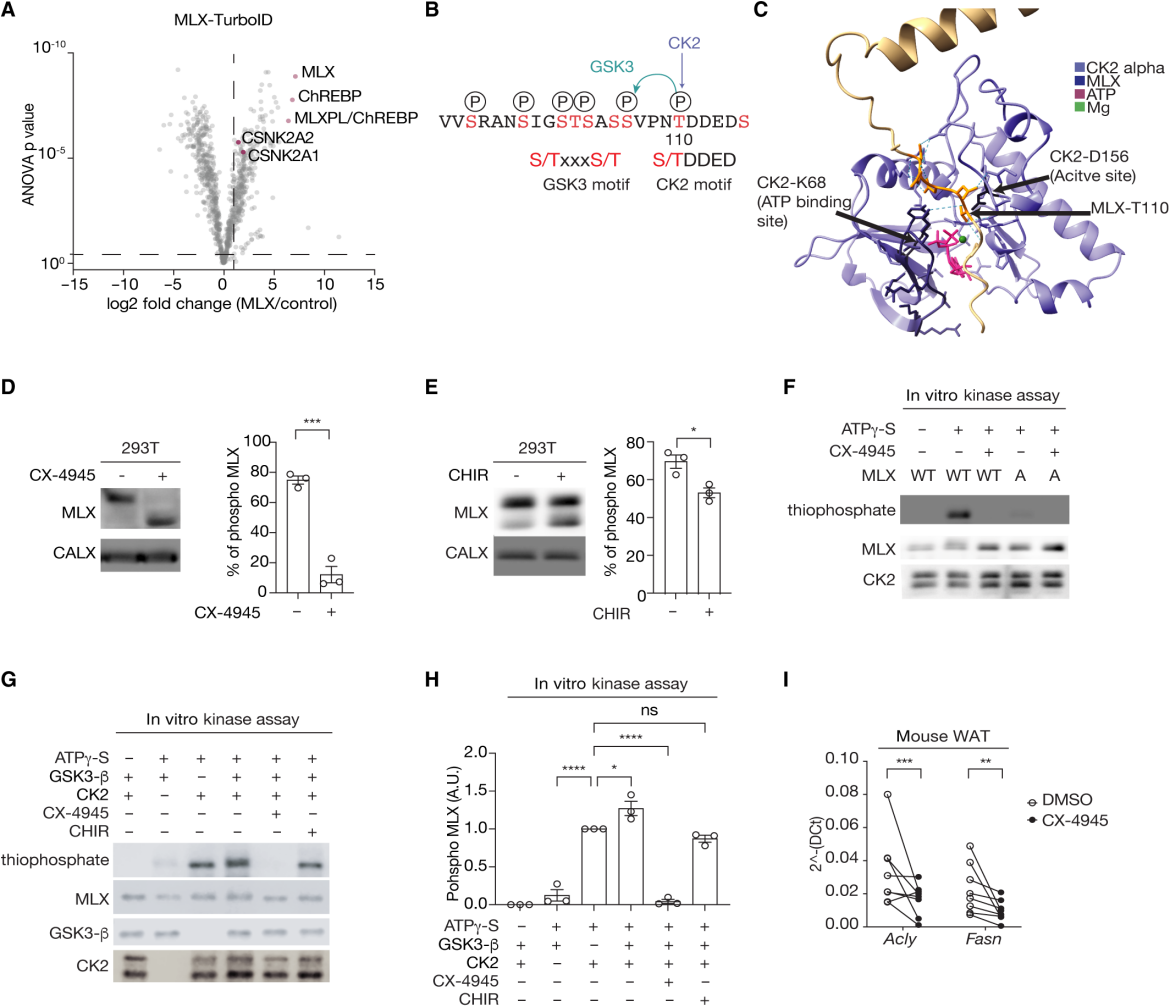
Identification of CK2 and GSK3 as MLX kinases. (**A**) Interactors of TurboID-tagged MLX. 293T cells expressing ChREBP, HK2, and MLX-TurboID were labeled with 25 nM biotin for 10 min. *N* = 4. (**B**) Putative CK2 and GSK3 phosphorylation motifs on MLX. (**C**) Interaction between MLX and the CK2 catalytic site based on an AlphaFold 3 structure prediction. (**D**) MLX phosphorylation in 293T cells treated with or without the CK2 inhibitor CX-4945 (10 μM) for 60 min. CALX serves as a loading control. *t* test, ***P < 0.001. *N* = 3. (**E**) MLX phosphorylation in 293T cells treated with or without the GSK3 inhibitor CHIR99021 (5 μM) for 90 min. *t* test, *P < 0.05. *N* = 3. (**F**) In vitro CK2-mediated MLX phosphorylation (thiophosphate). Recombinant CK2, MLX-WT or MLX-A, and γ-S were incubated with or without 25 nM CX-4945 for 30 min. *N* = 3. (**G** and **H**) In vitro CK2 and GSK3-mediated MLX phosphorylation (thiophosphate). Recombinant CK2, GSK3, MLX-WT, and ATPγ-S were incubated with or without 25 nM CX-4945 or 5 μM CHIR99021 for 30 min. One-way ANOVA, *P < 0.05 and ****P < 0.0001; ns, not significant. *N* = 3. (**I**) *Acly* and *Fasn* mRNA levels in mouse WAT explants treated with 10 μM CX-4945 for 60 min. *t* test, **P < 0.01 and ***P < 0.001. *n* = 5.

Next, we determined the upstream signaling pathway controlling MLX phosphorylation and the heterotetramer formation. Mammalian target of rapamycin complex 1 and 2 (mTORC1/2) are protein kinase complexes that promote ChREBP activity and de novo lipogenesis (DNL) (*26*–*28*), but mTOR inhibitors (rapamycin and torin2) had no impact on MLX phosphorylation (fig. S4A). To identify MLX kinases, we performed an unbiased and proteomics-based proximity ligation assay (*29*). By transiently expressing the MLX-TurboID fusion protein (fig. S4, B and C) in 293T cells, we searched for putative MLX kinases by mass spectrometry (MS). Among the enriched biotinylated kinases, CSNK2A1 and CSNK2A2, catalytic subunits of casein kinase 2 (CK2), were one of the top candidates (Fig. 4A). CK2 is known to phosphorylate the pS/T-D/E-X-D/E motif (*30*), found in the MLX phosphorylation site (Fig. 4B). An AlphaFold 3 (*25*) model indeed predicted that the CK2 catalytic site binds to the putative CK2 motif on MLX (Fig. 4C). Consistently, the adenosine 5′-triphosphate (ATP)–competitive CK2 inhibitor CX-4945 (*31*) inhibited MLX phosphorylation (Fig. 4D), suggesting that CK2 is an MLX kinase. CK2 has been shown to act as a priming kinase for GSK3 (*32*, *33*). The N-terminal region of the CK2 motif on the MLX phosphorylation site contains the consensus motif of GSK3 (pS/T-X-X-X-pS/T) (Fig. 4B). The GSK3 inhibitor CHIR99021 (Fig. 4E) (*34*) or genetic depletion of *GSK3* partially blocked MLX phosphorylation (fig. S4, D and E). These data suggest that CK2 and GSK3 are upstream kinases that control MLX phosphorylation.

To test whether CK2 and GSK3 directly phosphorylate MLX, we performed an in vitro CK2 and GSK3 kinase assay with recombinant MLX and ATP-γ-S as substrates (*35*, *36*). CK2 phosphorylated recombinant MLX-WT but not MLX-A (Fig. 4F). Although GSK3 alone very slightly phosphorylates MLX (Fig. 4, G and H), the combination of GSK3 with CK2 robustly increased MLX phosphorylation. CK2/GSK3-mediated MLX phosphorylation was completely blocked by CX-4945 but only partially blocked by CHIR99021 (Fig. 4G and 4H). On the basis of these data, we propose that CK2 phosphorylates MLX, which primes GSK3-mediated MLX phosphorylation (Fig. 4B). Consistent with this, the mutation of T110 and S115 in MLX to alanine was sufficient to block glucose-induced ChREBP-MLX activity, while alanine mutation on N-terminal serine/threonine (S86A, T87A, S91A, and S94A) had no impact on ChREBP-MLX activity (fig. S4, F and G), confirming that CK2-mediated MLX phosphorylation on T110 is the key phosphorylation for the ChREBP-MLX activity. To further test the role of CK2-mediated MLX phosphorylation, we treated mouse WAT explants with the CK2 inhibitor CX-4945 and examined the expression of ChREBP-MLX target genes (Fig. 4I). CX-4945 decreased expression of the ChREBP-MLX target genes *Acly* and *Fasn* in WAT. These data suggest that CK2 and GSK3 coordinately phosphorylate MLX, promoting ChREBP-MLX transcriptional activity.

### High G6P levels inhibit CK2-mediated MLX phosphorylation and the heterotetramer formation

**Fig. 5.**
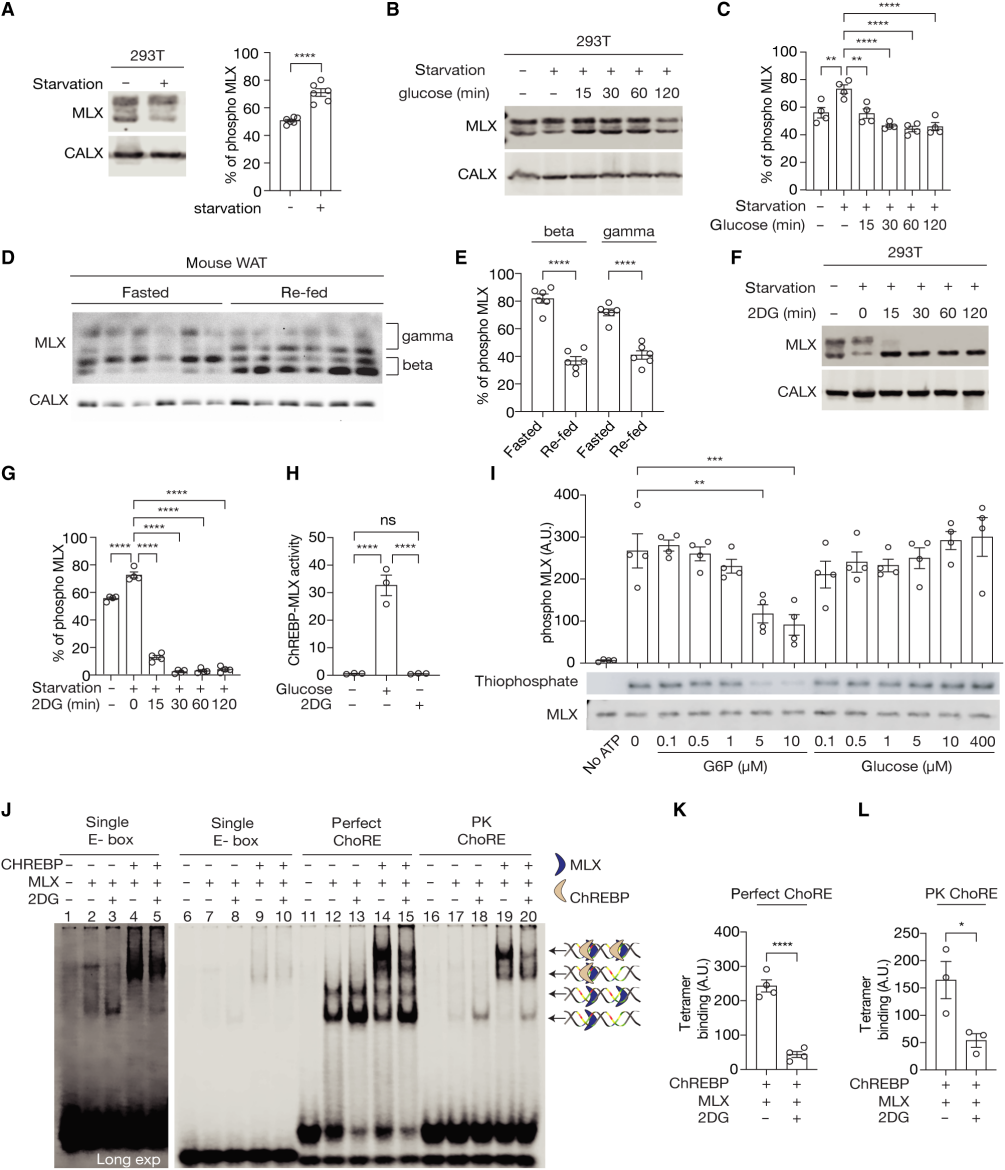
G6P accumulation inhibits CK2-mediated MLX phosphorylation and the binding of ChREBP-MLX tetramer to ChoRE. (**A**) MLX phosphorylation in 293T cells starved for glucose and serum. CALX serves as a loading control. t-test, ****P < 0.0001. *N* = 6. (**B** and **C**) MLX phosphorylation in 293T cells starved for glucose and serum overnight and re-fed with 25 mM glucose. One-way ANOVA, **P < 0.01 and ****P < 0.0001. *N* = 4. (**D** and **E**) MLX phosphorylation in WAT from overnight fasted or overnight fasted and 3-hour re-fed mice. One-way ANOVA, ****P < 0.0001. *n* = 6. (**F** and **G**) MLX phosphorylation in 293T cells starved for glucose and re-fed with 25 mM 2DG. One-way ANOVA, ****P < 0.0001. *N* = 4. (**H**) ChREBP-MLX luciferase reporter activity in 293T cells expressing ChREBP, HK2, and MLX-WT. Cells were starved for glucose and re-fed with 25 mM glucose or 25 mM 2DG for 3 hours. One-way ANOVA, ****P < 0.0001; ns, not significant. *N* = 3. (**I**) In vitro CK2-mediated MLX phosphorylation (thiophosphate) in the presence of different concentrations of glucose-6-phosphate (G6P) or glucose. One-way ANOVA, **P < 0.01 and ***P < 0.001. *N* = 4. (**J**) Binding of MLX and/or ChREBP to single E-box, perfect ChoRE, or PK ChoRE probes. ChREBP and MLX were purified from 293T cells treated with or without 25 mM 2DG for 1 hour. (**K** and **L**) Quantification of the data in (J). *t* test, *P < 0.05 and ****P < 0.0001. *N* = 3 to 4.

Since ChREBP-MLX activity is controlled by glucose (*3*), we wondered whether CK2/GSK3-mediated MLX phosphorylation is correlated with glucose availability. As the serum contains glucose, we examined MLX phosphorylation upon serum and glucose starvation. To our surprise, MLX phosphorylation increased in serum- and glucose-starved cells (Fig. 5A). Conversely, the addition of serum and glucose caused partial dephosphorylation of MLX (Fig. 5, B and C). To study the effects of feeding in vivo, we examined MLX phosphorylation in mouse liver and adipose tissue. MLX was phosphorylated in both tissues in fasted mice (Fig. 5D and fig. S5A). MLX phosphorylation decreased upon refeeding in WAT (Fig. 5, D and E), but not in the liver (fig. S5A), most likely due to the high expression of hepatic G6P phosphatase (*37*) (see below). These data suggest that MLX phosphorylation is inversely regulated by glucose availability in WAT.

These effects of glucose on MLX phosphorylation seem paradoxical to our earlier observation that MLX phosphorylation is required for the stability and activity of the ChREBP-MLX tetrameric complex and for the sugar response (Figs. 1F, 2E, and 3D, and fig. S1F). How can we explain this paradox? We hypothesized that in basal and starved conditions, MLX is phosphorylated, primed to be active. Upon an increase in glucose influx, glucose is phosphorylated to produce G6P, which in turn promotes ChREBP activity and thereby gene expression (*4*–*6*). As G6P levels increase, MLX phosphorylation is inhibited, thereby halting the ChREBP-MLX activity. Thus, glucose-induced MLX dephosphorylation may be a feedback mechanism to adjust ChREBP-MLX activity in response to glucose flux (fig. S5D) (*4*, *5*, *18*). To test this hypothesis, we treated cells with the glucose analog 2-deoxyglucose (2DG). Similar to glucose, 2DG is phosphorylated by hexokinases to produce the G6P analog 2DG-6-phosphate (2DG6P) (*38*). Unlike G6P, cells do not metabolize 2DG6P, and thus 2DG6P accumulates within cells, which in turn blocks glucose flux (*38*). 2DG inhibited MLX phosphorylation in a hexokinase-dependent manner (Fig. 5, F and G, and S5B). Unlike glucose (fig. S1D), 2DG did not promote ChREBP-MLX activity (Fig. 5H and fig. S5C). We further examined whether G6P directly inhibits CK2-mediated MLX phosphorylation by adding increasing concentrations of G6P in the in vitro CK2 kinase reaction. G6P, but not glucose, blocked CK2-mediated MLX phosphorylation at ≥5 μM, which is still the physiological G6P concentration range (*39*) (Fig. 5I). These results suggest that high levels of G6P directly inhibit CK2-mediated MLX phosphorylation. Consistent with the findings in phospho-deficient MLX-A mutant, MLX dephosphorylation by 2DG decreased the heterotetrametric binding of the ChREBP-MLX complex to the ChoRE sequence, but not the heterodimer binding to a single E-box (Fig. 5, J, K, and L; and fig. S5, E and K). These data suggest that G6P accumulation inhibits CK2-mediated MLX phosphorylation and thereby the formation of the ChREBP-MLX heterotetramer on the ChoRE.

## DISCUSSION

**Fig. 6.**
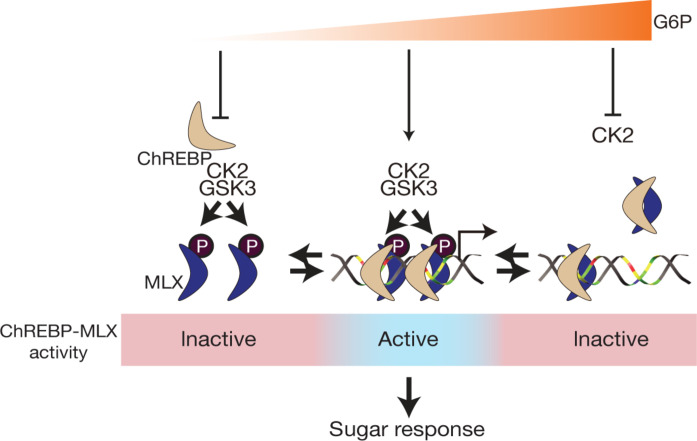
CK2/GSK3-mediated MLX phosphorylation stabilizes the heterotetrametric ChREBP-MLX complex on the ChoRE to promote the sugar response. Under basal and starvation conditions, CK2 and GSK3 phosphorylate MLX, priming it for activation. Following an increase glucose uptake, G6P activates ChREBP, which forms a stable heterotetrameric complex with phosphorylated MLX on the ChoRE. This stable binding promotes gene expression and the sugar response. As G6P levels increase, CK2-mediated MLX phosphorylation is inhibited, destabilizing the binding of the heterotetramer to the ChoRE and terminating the transcriptional response.

In the eukaryotic genome, an E-box is a key cis-regulatory element that controls gene expression. E-boxes, present approximately 15 million times in the human genome (*40*), are recognized by homo or heterodimers of the bHLH family of TFs (*41*). To date, more than 100 bHLH-containing family members have been identified in humans (*42*). How is the specificity of a bHLH TF for a specific E-box determined? In addition to the dimerization of particular bHLH proteins (*43*) and DNA sequences adjacent to the E-box (*44*), tandem E-boxes appear to be key elements conferring the target gene specificity for some bHLH TFs. Examples of such TFs include the heterotetramer of BMAL1-CLOCK (*45*), TWIST1-E proteins (*46*), and ChREBP/Mondo-MLX (*9*, *47*). To the best of our knowledge, the regulatory mechanisms controlling the formation of the abovementioned heterotetrameric bHLH complexes on tandem E-boxes are unknown. In this study, we show that CK2/GSK3-mediated MLX phosphorylation on an evolutionarily conserved motif stabilizes the ChREBP-MLX heterotetrametric complex on the sugar-responsive tandem E-boxes (Fig. 6). MLX phosphorylation is key for maintaining lipid storage and sugar tolerance in *Drosophila* (Fig. 2), highlighting the physiological importance of the ChREBP-MLX tetramer formation. We further demonstrate that G6P accumulation causes inhibition of CK2-mediated MLX phosphorylation, preventing the ChREBP-MLX tetramer formation on the ChoRE (Fig. 5). In summary, we propose that the CK2/GSK3-MLX signaling promotes the formation of ChREBP-MLX heterotetrametric complexes on the ChoRE, the ChREBP-MLX transcriptional activity, and thereby the sugar response. To our knowledge, this is the first study to reveal a signaling pathway that controls a bHLH tetrameric complex on tandem E-boxes in response to physiological cues.

How does MLX phosphorylation promote the ChREBP-MLX heterotetramer formation on the ChoRE? A model predicted by AlphaFold 3 revealed a conformational change in both ChREBP-MLX dimers on the ChoRE (Fig. 3B), which favors trans-interaction between the two dimers. Thus, MLX phosphorylation may be directly involved in the dimer-dimer interaction (Fig. 3B). Another possible explanation is that MLX phosphorylation promotes the interdimer interactions via the loop region of MLX since a previous model predicted that two MLX proteins face each other and the loop region of MLX forms interdimer interactions (*9*). However, the AlphaFold 3 model predicted that two ChREBP-MLX dimers bind tandem E-boxes in parallel, and we did not observe any interactions through the loop regions of MLX (Fig. 3B). However, when we modeled ChREBP-MLX heterotetramer with the MLX loop mutant used in Ma *et al.* (*9*), the interdimer interactions mediated by MLX phosphorylation were lost. These data may suggest that the loop region of MLX promotes interdimer interactions by bringing phosphate groups on MLX in proximity to ChREBP in the adjacent dimer. These models need to be further tested by cryo–electron microscopy structures.

MLX is known to act as a transcriptional activator or repressor, depending on its interaction partner (*48*). MLX interacts with several bHLH factors, including Max-binding protein MNT, Max dimerization proteins MXD1/MAD1 and MXD4/MAD4, and MondoA (encoded by *MLXIP*) (*7*, *15*, *48*–*50*). In our EMSA experiments, MLX-A displayed strong binding to the ChoRE sequence independently of ChREBP (Fig. 3D and fig. S3, G and H). Currently, the identity of the ChREBP-independent bands is unknown. In our Turbo-ID experiments, we could not detect the interaction of MLX with MNT, MXD1, and MXD4. MondoA has a similar molecular weight with ChREBP. Thus, if the ChREBP-independent binding was due to the MondoA-MLX-A complex, the mobility of MondoA-MLX-A should be similar to that of ChREBP-MLX in EMSA. Thus, it is unlikely that the ChREBP-independent binding is MondoA-MLX-A complex. Alternatively, the ChREBP-independent binding may correspond to an MLX monomer or homodimer (*15*). These possibilities need further investigation to fully understand the impact of MLX phosphorylation on the extended TF network beyond its role with ChREBP.

Our observation that G6P inhibits CK2-mediated MLX phosphorylation seems paradoxical to the known role of G6P in promoting ChREBP-MLX activity (*4*–*6*, *51*). How can G6P act as a positive and a negative regulator of the ChREBP-MLX complex? Glucose is an important carbon source for DNL through glycolysis. Glucose also provides reducing power for DNL by producing reduced form of nicotinamide adenine dinucleotide phosphate in the pentose phosphate pathway (PPP). G6P accumulation may reflect insufficient flux in glycolysis and the PPP to drive DNL. Thus, MLX dephosphorylation by G6P might serve as a feedback mechanism to couple expression of enzymes in DNL with glucose flux in glycolysis and the PPP. Alternatively, it is possible that different intercellular G6P pools activate and inhibit the ChREBP-MLX activity. Cytoplasmic G6P, directly coupled to the enzymatic activity of hexokinases, may promote the ChREBP-MLX activity, while noncytoplasmic G6P [e.g., nuclear G6P (*52*)] could inhibit the ChREBP-MLX tetramer formation on ChoRE. These two hypotheses are not mutually exclusive and need to be experimentally tested.

CK2 and GSK3 are highly conserved protein kinases in eukaryotes. Since mouse MLX was phosphorylated in *Drosophila* larvae, it is likely that fly Ck2 and Sgg (fly GSK3 ortholog) phosphorylate fly Mlx. MLX phosphorylation sites are absent in platyhelminths (flatworms) and nematodes (roundworms). These organisms synthesize lipids in the gut and lack a specialized organ to produce lipids from carbohydrates (e.g., liver-like and adipose tissue-like organs). Since MLX phosphorylation is regulated by glucose availability and is important for lipid storage, organisms with a specialized organ for lipid synthesis may have acquired the CK2/GSK3-MLX signaling pathway as an evolutionary strategy to better couple carbohydrate influx to lipid metabolism. Further investigations are necessary to identify the evolutionary origin and to reveal the significance of the CK2/GSK3-MLX pathway across different organisms.

CK2 is involved in various cellular functions including lipid metabolism (*30*, *53*). In the presence of insulin, CK2 promotes the activation of MED17, leading to the transcription of lipogenic genes via the TF USF1 (*54*). CK2 also promotes lipid synthesis through AKT-mTORC1 (*55*). Therefore, CK2 regulates the expression of lipogenic genes through multiple pathways including MLX phosphorylation.

Our findings may shed light on the pathogenesis of metabolic diseases. DNL in the liver and adipose tissue plays a crucial role in maintaining whole-body glucose homeostasis, diabetes, metabolic dysfunction–associated steatotic liver disease, and liver cancer (*56*–*58*). Furthermore, MLX also plays a pivotal role in MYC-induced tumorigenesis (*59*, *60*), underscoring the importance of MLX function in tumor development. Our findings that GSK3 and CK2 are MLX kinases may suggest that targeting these kinases might be effective therapeutics for the above-mentioned metabolic diseases.

## MATERIALS AND METHODS

### Human samples

Omental WAT (oWAT) biopsies were obtained from lean subjects with a normal fasting glucose level and body mass index <27 kg/m^2^ (*18*). All subjects gave informed consent before the surgical procedure. Patients were fasted overnight and underwent general anesthesia. All oWAT specimens were obtained between 8:30 and 12:00 a.m., snap-frozen in liquid nitrogen, and stored at −80°C for subsequent use. The study protocol was approved by the Ethikkomission Nordwest- und Zentralschweiz (EKNZ, BASEC 2016-01040).

### Animals

C57BL/6 mice were purchased from an internal stock from the KU Leuven animal facility and epididymal WAT was collected. For analysis of MLX phosphorylation, WAT samples were snap-frozen and kept at −80°C for subsequent use. For the explant experiment, WATs were washed with phosphate-buffered saline (PBS) and cultured in Dulbecco’s modified Eagle’s medium (DMEM) high glucose (Thermo Fisher Scientific, 41965062) supplemented with 1 mM sodium pyruvate (Thermo Fisher Scientific, 11360039), 1× penicillin and streptomycin (Sigma-Aldrich, P4333), and 10% fetal bovine serum (FBS; Thermo Fisher Scientific, 10270106 lot no. 2319372) and treated with 10 μM CX-4945 (Biorbyt, ORB180973) for 60 min. The tissues were washed with ice-cold PBS, snap-frozen, and stored at −80°C for subsequent use. The study protocol was approved by the KU Leuven animal ethical committee (#206/2020).

### Cell culture

293T cells were obtained from G. Carmeliet (KU Leuven) and 3T3 L1 cells were obtained from the American Type Culture Collection. Cells were cultured in M1 medium composed of DMEM high glucose (Thermo Fisher Scientific, 41965062) supplemented with 1 mM sodium pyruvate (Thermo Fisher Scientific, 11360039), 1× penicillin and streptomycin (Sigma-Aldrich, P4333), and 10% FBS (Thermo Fisher Scientific, 10270106 lot no. 2319372). Cells were cultured in a 37°C incubator with 5% CO_2_. For starvation, cells were washed twice with starvation media (DMEM without glucose; Thermo Fisher Scientific, 11966025) supplemented with 1 mM sodium pyruvate (Thermo Fisher Scientific, 11360039), 1× penicillin and streptomycin (Sigma-Aldrich, P4333) and kept in starvation media. For GSK3, knockout/knockdown cells were generated by transfecting 293T cells with the corresponding lentiCRISPR version 2 plasmid with jetPRIME (Polyplus-transfection, 101000046) and selected with puromycin (InvivoGen ant-pr-1) (1 μg/ml) for 2 days followed by transfection of GSK3-β siRNA (Integrated DNA Technologies Inc., design ID: hs.Ri.GSK3B.13.1, hs.Ri.GSK3B.13.2, and hs.Ri.GSK3B.13.3). For overexpression experiments in 293T cells, unless otherwise stated, cells were transfected with plasmids harboring genes encoding mouse ChREBP, rat HK2, and mouse MLX with jetPRIME (Polyplus-transfection, 101000046). For the cycloheximide chase experiment, 293T cells were treated with 50 μM cycloheximide (Sigma-Aldrich, C4859). 293T cells were treated with 100 nM rapamycin [LC Laboratories, R-5000 in dimethyl sulfoxide (DMSO)], 5 μM CHIR99021 (Sigma-Aldrich, SML1046 in DMSO), or 250 nM torin2 (LC Laboratories, T-8448 in DMSO) for 90 min or with 10 μM CX-4945 (Biorbyt, ORB180973 in DMSO) for 60 min. 3T3 L1 cells were cultured and differentiated as previously described (*18*). For differentiation, cells were maintained in M1 medium for 2 days after reaching confluence, followed by 2-day treatment with M2 medium composed of M1 medium supplemented with insulin (1.5 μg/ml; Sigma-Aldrich, I9278), 0.5 mM 3-isobutyl-1-methylxanthine (AdipoGen LIFE SCIENCES, AG-CR1-3512-G001), 1 μM dexamethasone (Sigma-Aldrich, D4902), and 2 μM rosiglitazone (AdipoGen LIFE SCIENCES, AG-CR1-3570), followed by a 4-day incubation with M3 medium [M1 with insulin (1.5 μg/ml)]. Differentiated cells were maintained in M3 medium, which was changed every 2 days.

### Plasmids

For protein expression, cDNA was cloned and inserted into pcDNA3 for mammalian expression, pGEX for bacterial expression, or pUAST for fly experiments by using the primers listed in table S1. Mutations were introduced by reverse polymerase chain reaction (PCR; table S1), followed by Dpn I (New England Biolabs, R0176L) digestion and bacterial transformation. For GSK3 KO, sgRNAs targeting GSK3α (table S1) were cloned and inserted into lentiCRISPRv2 (a gift from F. Zhang) as previously described (*61*). The plasmid sequences were validated.

### Immunoblots

Tissues or cells were lysed in a lysis buffer [100 mM tris (pH 7.5; Sigma-Aldrich, T1503), 2 mM EDTA (Thermo Fisher Scientific, 10522965), 2 mM EGTA (Sigma-Aldrich E4378), 150 mM NaCl (Fisher Chemical, S/3160/60), 1% Triton X-100 (Fisher Chemical, T/3751/08), cOmplete Mini EDTA-free Protease I (Roche, 469315900), and PhosSTOP (Roche, 4906837001)]. For tissue samples, protein concentration was determined by Bradford assay (Bio-rad, 5000006). Equal amounts of proteins were separated by SDS–polyacrylamide gel electrophoresis (PAGE) and transferred onto nitrocellulose membranes (Amersham Protran Premium Western blotting membranes, GE10600003). Membranes were blocked with Interceptor reagent (Li-COR, 927-70001) and antibodies were diluted with 3% bovine serum albumin (Thermo Fisher Scientific, BP9703-100). The antibodies used in this study are listed in table S2. Images were captured with a Li-COR Odyssey XF. Quantification was performed with ImageJ software [National Institutes of Health (NIH)].

### CIP treatment

Protein lysates from mouse WAT or 293T cells transiently expressing ChREBP, MLX, and HK2 were incubated with 5 μl of CIP (Quick CIP New England Biolabs, M0525S) and/or PhosSTOP (Roche, 4906837001) in 40 μl of 1× CutSmart buffer (New England Biolabs, B6004S). The reaction was incubated at 37°C for 30 min with shaking at 800 rpm. The reaction was stopped by adding 12.5 μl of 5× SDS sample buffer, and the samples were incubated at 65°C for 15 min before immunoblotting.

### Luciferase assay

293T cells were transfected with pGL3-ChoRE-Luc plasmid, pNL1.1-Nluc/TK plasmid, and plasmids described in figure legends and table S3. Cells were starved for glucose for 16 hours and re-fed with 25 mM glucose or 2DG for 3 hours. Luciferase activity was measured with Nano-Glo Dual-Luciferase Reporter Assay System (Promega, N1521) according to the manufacturer’s instructions.

### Fly stocks and husbandry

Flies were maintained at 25°C in a 12-hour light/12-hour dark cycle, on standard *Drosophila* medium [0.6% agar (w/v), 3.2% semolina (w/v), 6.5% malt (w/v), 1.8% dry baker’s yeast (w/v), 0.7% propionic acid (v/v), and 2.4% Nipagin (methylparaben) (v/v)]. Experiments were conducted on a diet containing 10% dry baker’s yeast (w/v), 0.5% agarose (w/v), 0.7% propionic acid (v/v), and 2.5% Nipagin (v/v), supplemented with 5 or 15% sucrose (w/v) for MSD or HSD, respectively.

The following lines were generated in this study: *UAS-mMlx-WT* and *UAS-mMlx-A*. Wild-type mouse *Mlx* and the mutant version were cloned and inserted into the pUAST-attB vector and directed to the attP2 landing site on chromosome 3. Transgenic flies were generated by WellGenetics Inc. and recombined with the *mlx*-null mutant flies, *mlx^1^* (*10*). As a control, we recovered lines from which the P-element had been excised precisely, leaving *mlx* intact (*10*). The following genotypes of larvae were used in the analyses: FB-Gal4; Control, FB-Gal4; *mlx^1^*, FB-Gal4; *UAS-mMlx-WT*, *mlx^1^*, and FB-Gal4; *UAS-mMlx-A*, *mlx^1^.*

### Fly pupariation and growth analysis

Flies were allowed to lay eggs for 24 hours on apple juice plates [33.33% apple juice (v/v), 1.75% agar (w/v), 2.5% sugar (w/v), and 2.0% Nipagin (v/v)], supplemented with dry yeast, kept at 25°C. After 24 hours, 30× first instar larvae were collected to vials containing yeast or sugar diets and kept at 25°C. Pupariation was scored every 24 hours, and survival was recorded at the end of the observation period.

### LipidTOX staining

Fat bodies from early third instar larvae were fixed in 4% formaldehyde for 30 min and washed three times with PBS. Fat bodies were stained with LipidTOX (Thermo Fisher Scientific, H34477) 1:400 in PBS for 30 min at room temperature, washed three times with PBS, and mounted using VECTASHIELD Mounting Medium with DAPI (VectorLabs, H-1200). Samples were imaged using a Leica SP8 upright microscope, and images were processed using Imaris software (Oxford Instruments).

### LipidTOX image quantification

For lipid droplet analysis, the cell borders were defined in multiple z layers using the Imaris custom surface tool. Lipid staining was masked within the rendered cell surface to have lipids within cell borders. Surface detection tool was used on the masked lipid staining to determine the total lipid volume within the cell. The lipid volume was normalized to the cell volume.

### Fly TAG analysis

For each replicate, 10× early third instar larvae per group were collected and snap frozen in liquid nitrogen. Larvae were homogenized in 300 μl of cold 1× PBS (+0.05% Tween 20), and the lysates were inactivated at 70°C for 10 min. Glycerol and TAG levels were measured by using the coupled colorimetric assay kits (Free Glycerol kit, Cayman Chemical, 10010755; and Triacyl glyceride kit, Cayman Chemical, 10010303). The values were normalized to body weight.

### Fly sugar analysis

Hemolymph from third instar larvae was extracted as described previously (*62*) and the circulating glucose and trehalose levels were measured with the Glucose HK assay reagent (Sigma-Aldrich, GAHK-20) and trehalase from porcine kidney (Sigma-Aldrich, T8778-1U) as described previously (*63*).

### BioID

293T cells were transfected with plasmids harboring genes encoding ChREBP, HK2, and TurboID-tagged MLX. Twenty-four hours after transfection, cells were treated with 25 nM biotin for 10 min. Cells were lysed in lysis buffer [100 mM tris (pH 7.5; Sigma-Aldrich, T1503), 2 mM EDTA (Thermo Fisher Scientific,10522965), 2 mM EGTA (Sigma-Aldrich, E4378), 150 mM NaCl (Fisher Chemical S/3160/60), 1% Triton X-100 (Fisher Chemical, T/3751/08), cOmplete Mini EDTA-free Protease I (Roche, 469315900), and PhosSTOP (Roche, 4906837001)]. Biotinylated proteins were pulled down by streptavidin-coupled magnetic beads (Thermo Fisher Scientific) according to (*29*). Proteins were precipitated with trichloroacetic acid, alkylated, and digested with modified trypsin (enzyme/protein ratio, 1:50) overnight. Peptides were desalted using C18 reverse-phase spin columns (Macrospin, Harvard Apparatus) according to the manufacturer’s instructions, dried under vacuum, and stored at −20°C until further use.

### Proteomics

The digested samples were injected (5 μl) and separated on an Ultimate 3000 UPLC system (Dionex, Thermo Fisher Scientific) equipped with an Acclaim PepMap100 pre-column (C18 particle size of 3 μm, pore size of 100 Å, diameter of 0.075 mm, and length of 20 mm; Thermo Fisher Scientific) and a C18 PepMap RSLC (particle size of 2 μm, pore size of 100 Å, diameter of 50 μm, and length of 150 mm; Thermo Fisher Scientific) using a linear gradient (0.300 μl/min). The composition of buffer A is pure water containing 0.1% formic acid. The composition of buffer B is pure water containing 0.08% formic acid and 80% acetonitrile. The fraction of buffer B increased from 0 to 4% in 3 min, from 4 to 10% in 12 min, from 10 to 35% in 20 min, from 35 to 65% in 5 min, from 65 to 95% in 1 min, and stayed at 95% for 10 min. The fraction of buffer B decreased from 95 to 5% in 1 min and stayed at 5% for 10 min. The Q Exactive Orbitrap mass spectrometer (Thermo Fisher Scientific) was operated in positive ion mode with a nanospray voltage of 2.1 kV and a source temperature of 250°C. Pierce LTQ Velos ESI positive ion calibration mix (Thermo Fisher Scientific, 88323) was used as an external calibrant. The instrument was operated in data-dependent acquisition mode with a survey MS scan at a resolution of 70,000 [full width at half maximum at mass/charge ratio (*m*/*z*) of 200] for the mass range of *m*/*z* 400 to 1600 for precursor ions, followed by MS/MS scans of the top 10 most intense peaks with +2, +3, +4, and +5 charged ions above a threshold ion count of 1 × 10^6^ at 17,500 resolution using normalized collision energy of 25 eV with an isolation window of 3.0 *m*/*z*, Apex trigger of 5 to 15 s, and dynamic exclusion of 10 s. All data were acquired with Xcalibur 3.1.66.10 software (Thermo Fisher Scientific). Peptide and protein quantities were computed with the software Progenesis (Nonlinear Dynamics). For protein identification, the spectra were exported and submitted to the protein identification software Mascot (version 2.2.06; Matrix science, London, England) and checked against all entries present an in house UniProt-based data base where ChREBP, MLX turbo, and rnHK2 were added as additional entries. Spectra were searched with a mass tolerance of 10 parts per million on the precursor mass and 0.02 Da on the fragments. Tolerated variable modifications were oxidation of M and deamidation of NQ and fixed modification for carbamidomethyl C. Tolerated miscleavages was set to 2. Mascot results were imported back in Progenesis (Nonlinear Dynamics).

### Purification of GST-MLX fusion protein

BL21 cells (New England Biolabs, C2527H) harboring pGEX-MLX-WT or pGEX-MLX-A plasmids were cultured in LB media to an optical density = 0.6 at 30°C, and the expression of recombinant MLX was induced by 0.25 mM of isopropyl-β-d-thiogalactopyranoside (Thermo Fisher Scientific, R1171) for 5 hours. Bacteria were lysed with a lysis buffer [20 mM HEPES (pH 7.5; VWR, 30487.297), 2 mM EDTA (Thermo Fisher Scientific, 10522965), 100 mM NaCl (Thermo Fisher Scientific, S/3160/60), 2 mM β-mercaptoethanol, cOmplete Mini EDTA-free Protease I (Roche, 4693159001), and 1% Triton X-100 (Thermo Fisher Scientific, T/3751/08)] with sonication for 4× 30 s on ice. Glutathione *S*-transferase (GST) fusion proteins were purified using glutathione agarose beads (Thermo Fisher Scientific, 16100) and eluted with glutathione (Sigma-Aldrich, G4251-10G).

### In vitro kinase assay

In vitro kinase reactions were performed in 40 μl of solution consisting of 1× NEBuffer for protein kinases (PK) (New England Biolabs, B6022SVIAL), 2 mM ATP-γ-S kinase substrate (Abcam, ab138911), 1 U (100 nM) CK2 (New England Biolabs, P6010SVIAL) and/or recombinant human GSK3-β (Abcam, ab63193). After incubation at 30°C for 30 min, 30 μl of the reaction mixture was alkylated with 1.5 μl of 50 mM p-nitrobenzyl mesylate and alkylation reagent (Abcam, ab138910) for 1 hour at room temperature. The reaction was stopped by adding 6 μl of 5× SDS sample buffer, and the samples were incubated at 65°C for 10 min. The proteins were separated by SDS-PAGE and transferred to nitrocellulose membranes for immunodetection. Membranes were blocked with Interceptor reagent (LiCOR). Phosphorylated MLX was detected with anti-thiophosphate ester antibody (Abcam, ab92570) and secondary IRDye 800CW Goat anti-rabbit immunoglobulin G (LI-COR Biotech 926-32211).

### RNA isolation and RT-PCR

Total RNA was isolated from mouse WAT and cultured cells with TRIzol reagent (Sigma-Aldrich) and NucleoSpin RNA (M&N, 740955-250), followed by cDNA synthesis using iScript cDNA synthesis kit (Bio-Rad, 1708891). Semiquantitative real-time PCR (RT-PCR) analysis was performed using fast SYBR green (Applied Biosystems, 10631376) on a StepOnePlus Real-Time PCR System (Applied Biosystems). Relative expression levels were determined by normalizing to S18 expression using the ΔΔCT method. The sequences for the primers used in this study can be found in the table S1.

### Electrophoretic mobility shift assay (EMSA)

Protein lysates (1 to 2 mg) of 293T cells overexpressing FLAG-tagged MLX alone or FLAG-tagged ChREBP and FLAG-tagged MLX were immunoprecipitated for 2.5 hours at 4°C with 25 μl of Anti-FLAG M2 Affinity Gel (Sigma-Aldrich, A2220). Samples were eluted with 3X FLAG peptide (0.2 μg/ml; Sigma-Aldrich, F4799). Eluted proteins were used for the Odyssey EMSA assay (829-07910). In brief, a 20-μl reaction mixture was prepared using 5 μl of purified protein lysate, 1 μl of 50 nM IRD800 5′ end–labeled aligned DNA probe, 2 μl of 25 mM dithiothreitol (DTT)/2.5% Tween 20, poly (dI•dC) (1 μg/μl), and 1 μl of 50% glycerol in 1× binding buffer [10 mM tris (pH 7.5; Sigma-Aldrich, T1503), 50 mM KCl, and 1 mM DTT]. For validation of the bound proteins, 1 μl of anti-MLX (Cell Signaling Technologies, 85570) or anti-ChREBP antibodies (Cell Signaling Technologies, 58069) was added. Samples were separated in a pre-run 4.5% native polyacrylamide gel in 0.5× TBE + 2.5% glycerol buffer at 50 V for 135 min. The images were captured with a Li-COR Odyssey XF. Quantification was performed using ImageJ software (NIH).

### AlphaFold prediction

AlphaFold 3 prediction was performed with Google DeepMind using human CSNK2A1, MLX-gamma sequence, and ChREBP with the PK ChoRE sequence (gggcgCGCGTGgagctCAGGTGgttcc). Models were analyzed by UCSF Chimera X (*64*).

### Alignment

For conservation analysis, MLX protein sequences and alignments were obtained from the UniProt database (https://uniprot.org/). Phylogenic analysis was performed with (https://ebi.ac.uk/jdispatcher/phylogeny/simple_phylogeny) (*65*).

### Statistical analysis

All data are shown as the mean ± SEM. Sample numbers are indicated in each figure legend. *n* represents the independent biological replicates, and *N* represents the number of independent experiments. To determine the statistical significance, *t* test for two comparisons, one-wayt analysis of variance (ANOVA) for multiple comparisons, and two-way ANOVA for groups analysis were performed with GraphPad Prism 9 (GraphPad Software). *P* values are indicated in each figure.

## Supplementary Materials

### This PDF file includes:

Figs. S1 to S5 Tables S1 to S3 References
