# Supplementary material for: MLX phosphorylation stabilizes the ChREBP-MLX heterotetramer on tandem E-boxes to control carbohydrate and lipid metabolism
Source: Sci Adv. 2025 Mar 12;11(11):eadt4548. doi: 10.1126/sciadv.adt4548 (PMC11900861; doi:10.1126/sciadv.adt4548)
Supplement: Supplementary file 1 — Figs. S1 to S5 Tables S1 to S3 References [file sciadv.adt4548_sm.pdf]

Science Advances
AAAS

Supplementary Materials for

MLX phosphorylation stabilizes the ChREBP-MLX heterotetramer on tandem E-boxes to control carbohydrate and lipid metabolism

Carla E. Cadena del Castillo *et al.*

Corresponding author: Mitsugu Shimobayashi, mitsugu.shimobayashi@kuleuven.be

Sci. Adv. **11**, eadt4548 (2025)
DOI: 10.1126/sciadv.adt4548

This PDF file includes:

Figs. S1 to S5
Tables S1 to S3
References

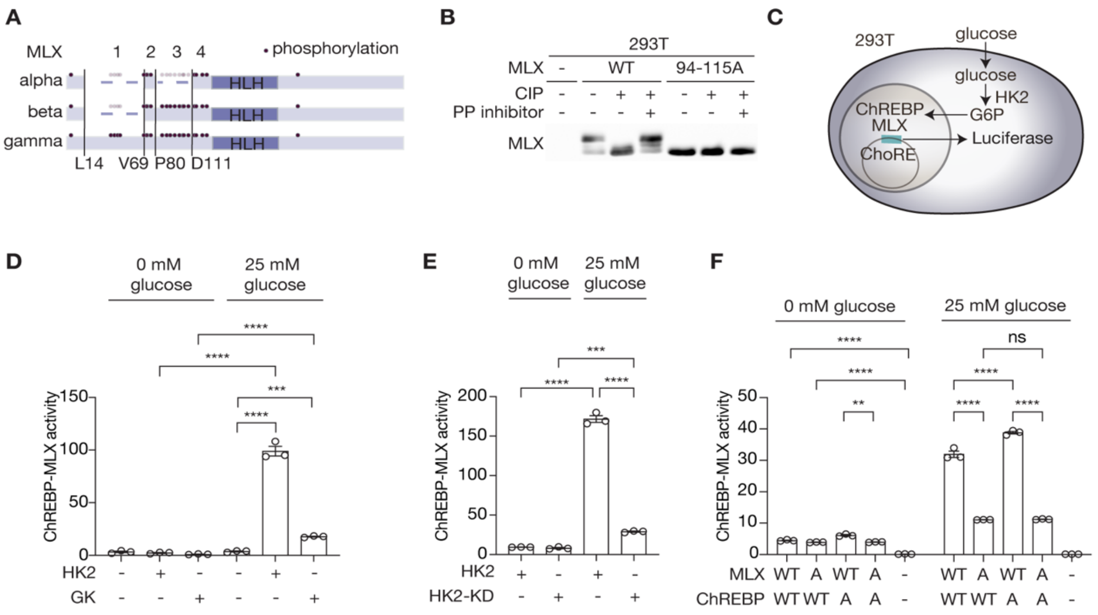

Fig. S1. MLX phosphorylation promotes ChREBP-MLX activity, related to Fig. 1.

A. Phosphorylation sites on MLX alpha, beta, or gamma isoforms according to PhosphoSitePlus® (https://phosphosite.org). **B**. MLX phosphorylation in 293T cells expressing MLX-WT or -A. Protein lysates were treated with calf intestine phosphatase (CIP) or CIP and phosphatase (PP) inhibitors. N=3. **C**. Luciferase reporter assay for ChREBP-MLX activity. **D**. ChREBP-MLX luciferase reporter activity in 293T cells expressing ChREBP, MLX-WT, and either HK2 or GCK. Cells were starved for glucose and treated with 25 mM glucose for 3 hours. One-way ANOVA, ***p<0.001, ****p<0.0001. N=3. **E**. ChREBP-MLX luciferase reporter activity in 293T cells expressing ChREBP, MLX-WT, and either HK2 or the kinase dead HK2 (HK2-KD). Cells were starved for glucose and treated with 25 mM glucose for 3 hours. One-way ANOVA, ***p<0.001, ****p<0.0001. N=3. **F**. ChREBP-MLX luciferase reporter activity in 293T cells expressing HK2, MLX-WT or -A and either ChREBP-WT or -A. Cells were starved for glucose and treated with 25 mM glucose for 3 hours. One-way ANOVA, **p<0.01, ****p<0.0001, ns=not significant. N=3.

A

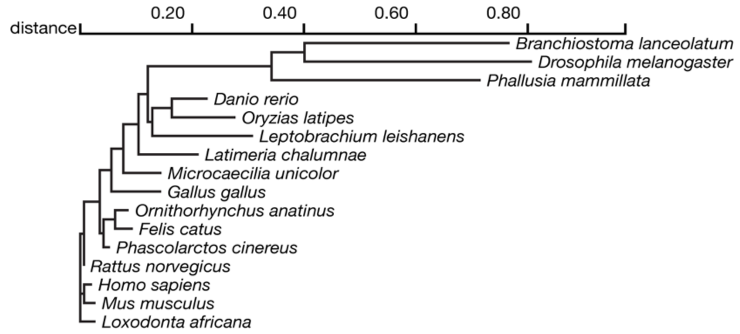

B

Branchiostoma lanceolatum	100.00	45.32	44.78	47.59	47.30	40.30	43.15	49.31	45.89	46.48	49.32	43.41	43.96	39.13	42.62	47.97
Drosophila melanogaster	45.32	100.00	42.41	41.99	43.29	42.54	41.30	43.17	44.54	43.56	43.23	44.10	44.10	44.10	44.10	45.71
Phallusia mammillata	44.78	42.41	100.00	45.85	45.85	46.70	49.12	47.32	44.93	49.55	47.35	46.70	46.70	47.11	46.26	48.29
Danio rerio	47.59	41.99	45.85	100.00	81.38	73.25	78.42	76.05	74.69	77.54	78.33	74.59	75.00	78.15	74.18	78.50
Oryzias latipes	47.30	43.29	45.85	81.38	100.00	68.83	73.47	70.66	70.20	71.67	71.72	71.77	71.77	74.37	70.56	75.70
Leptobrachium leishanens	40.30	42.54	46.70	73.25	68.83	100.00	71.49	75.21	72.84	73.53	75.21	63.39	62.71	66.55	62.24	78.76
Latimeria chalumnae	43.15	41.30	49.12	78.42	73.47	71.49	100.00	78.51	75.41	78.33	78.69	76.23	76.23	79.41	75.82	79.44
Microcaecilia unicolor	49.31	43.17	47.32	76.05	70.66	75.21	78.51	100.00	80.58	82.77	84.30	82.64	82.64	86.02	81.82	85.38
Gallus gallus	45.89	44.54	44.93	74.69	70.20	72.84	75.41	80.58	100.00	80.83	85.25	82.86	82.45	84.45	81.63	85.98
Ornithorhynchus anatinus	46.48	43.56	49.55	77.54	71.67	73.53	78.33	82.77	80.83	100.00	90.83	89.17	90.00	93.59	88.75	91.43
Phascolarctos cinereus	49.32	43.23	47.35	78.33	71.72	75.21	78.69	84.30	85.25	90.83	100.00	91.39	91.80	95.38	91.39	95.33
Loxodonta africana	43.41	44.10	46.70	74.59	71.77	63.39	76.23	82.64	82.86	89.17	91.39	100.00	95.27	84.75	92.86	96.90
Homo sapiens	43.96	44.10	46.70	75.00	71.77	62.71	76.23	82.64	82.45	90.00	91.80	95.27	100.00	84.40	95.95	97.37
Felis catus	39.13	44.10	47.11	78.15	74.37	66.55	79.41	86.02	84.45	93.59	95.38	84.75	84.40	100.00	83.69	92.98
Mus musculus	42.62	44.10	46.26	74.18	70.56	62.24	75.82	81.82	81.63	88.75	91.39	92.86	95.95	83.69	100.00	98.68
Rattus norvegicus	47.97	45.71	48.29	78.50	75.70	78.76	79.44	85.38	85.98	91.43	95.33	96.90	97.37	92.98	98.68	100.00

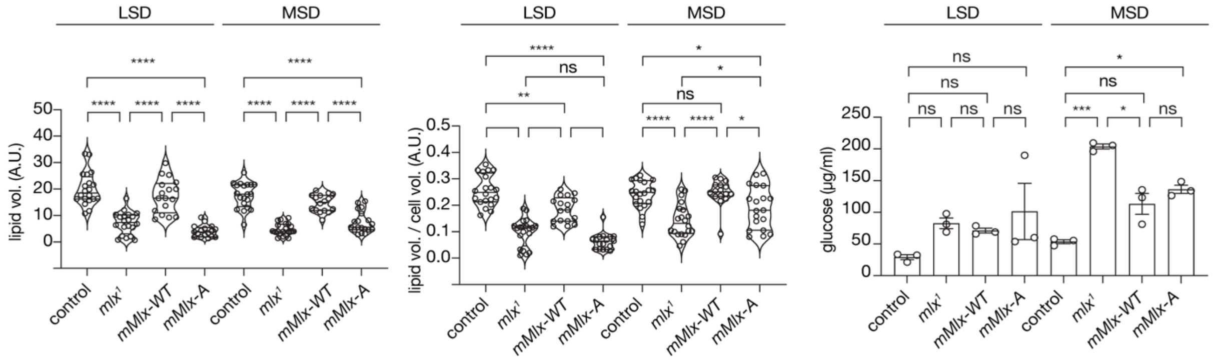

Fig. S2. MLX phosphorylation on an evolutionarily conserved motif promotes the sugar response in *Drosophila*, related to Fig. 2.

A. Evolutionary relationships of the MLX sequence among animal species. The branching order shows the relationships between species and branch length displays the amount of evolutionary change between the nodes. **B.** Percentage identity matrix of the MLX sequence among animal species. **C**. Quantification of lipid staining in Fig. 2G. Each point represents lipid signals per cell. Two-way ANOVA, ****p<0.0001. n=5. **D**. Quantification of lipid staining in Fig. 2G. Each point represents lipid signals normalized by cell volume. Two-way ANOVA, *p<0.05, **p<0.01, ****p<0.0001, ns=not significant. n=5. **E**. Hemolymph glucose levels in 3rd instar control, *mlx[1]*,

mMlx-WT or *mMlx-A* grown in LSD or MSD-fed conditions. Two-way ANOVA, *$p<0.05$, ***$p<0.001$, ns=not significant. N=3.

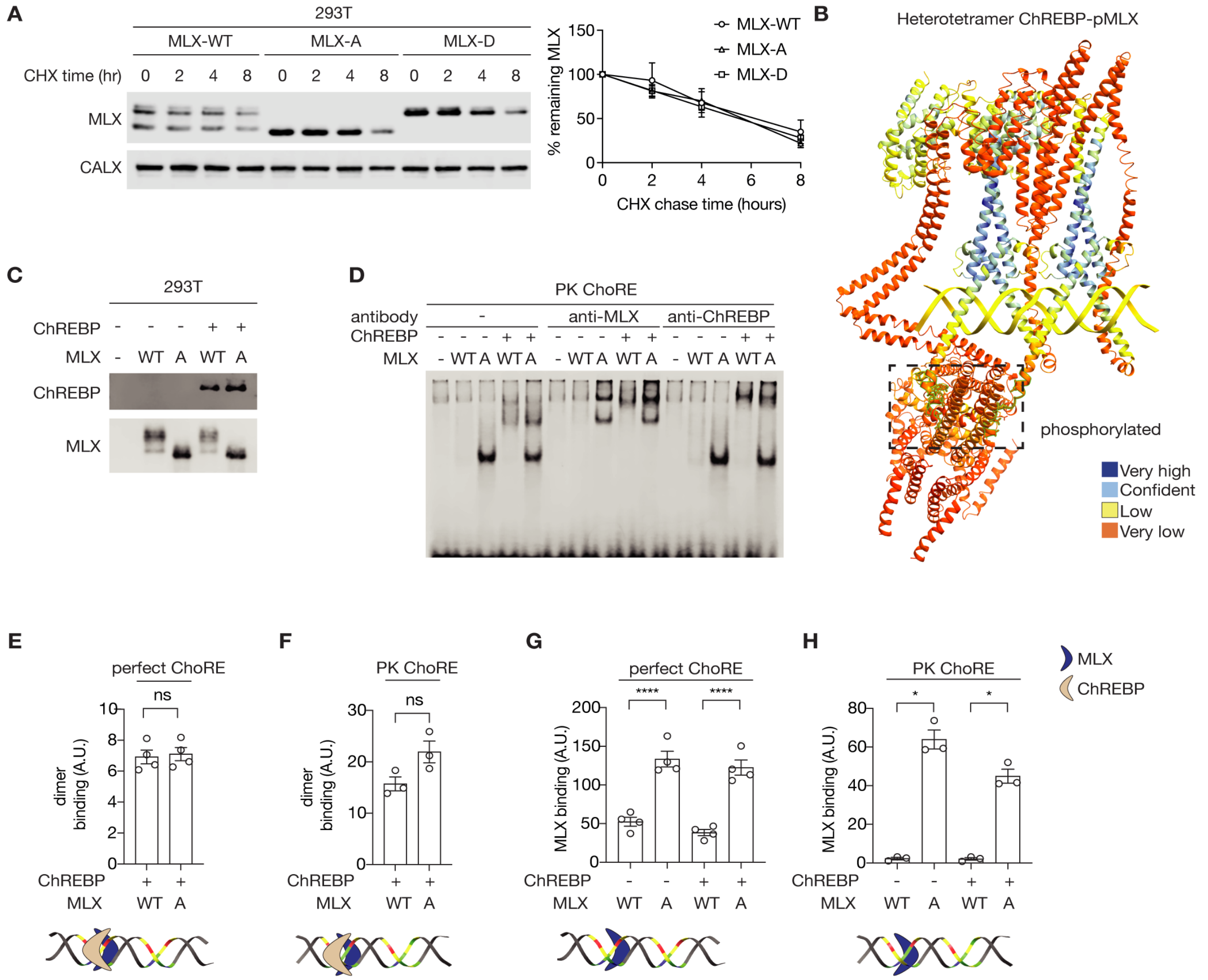

Fig. S3. MLX phosphorylation is required for the binding of ChREBP-MLX heterotetrametric complex to the ChoRE, related to Fig. 3.

A. MLX protein stability in 293T cells expressing MLX-WT -A, or -D. Cells were treated with 50 µM cycloheximide (CHX) for the indicated time. N=4. **B**. Model of ChREBP-phosphorylated MLX (pMLX) structure by AlphaFold 3 with confidence scores. **C**. Immunoprecipitated MLX and ChREBP proteins from in 293T cells expressing MLX-WT or -A with or without ChREBP for Electrophoretic mobility shift assay (EMSA) experiments in Fig. 3D. **D.** EMSA with antibodies against ChREBP or MLX validating the band identities shown in Fig. 3D and 5J. **E-H.** Quantification of the data in Fig. 3D. t-test, *p<0.05, ****p<0.0001, ns=not significant. N=3-4.

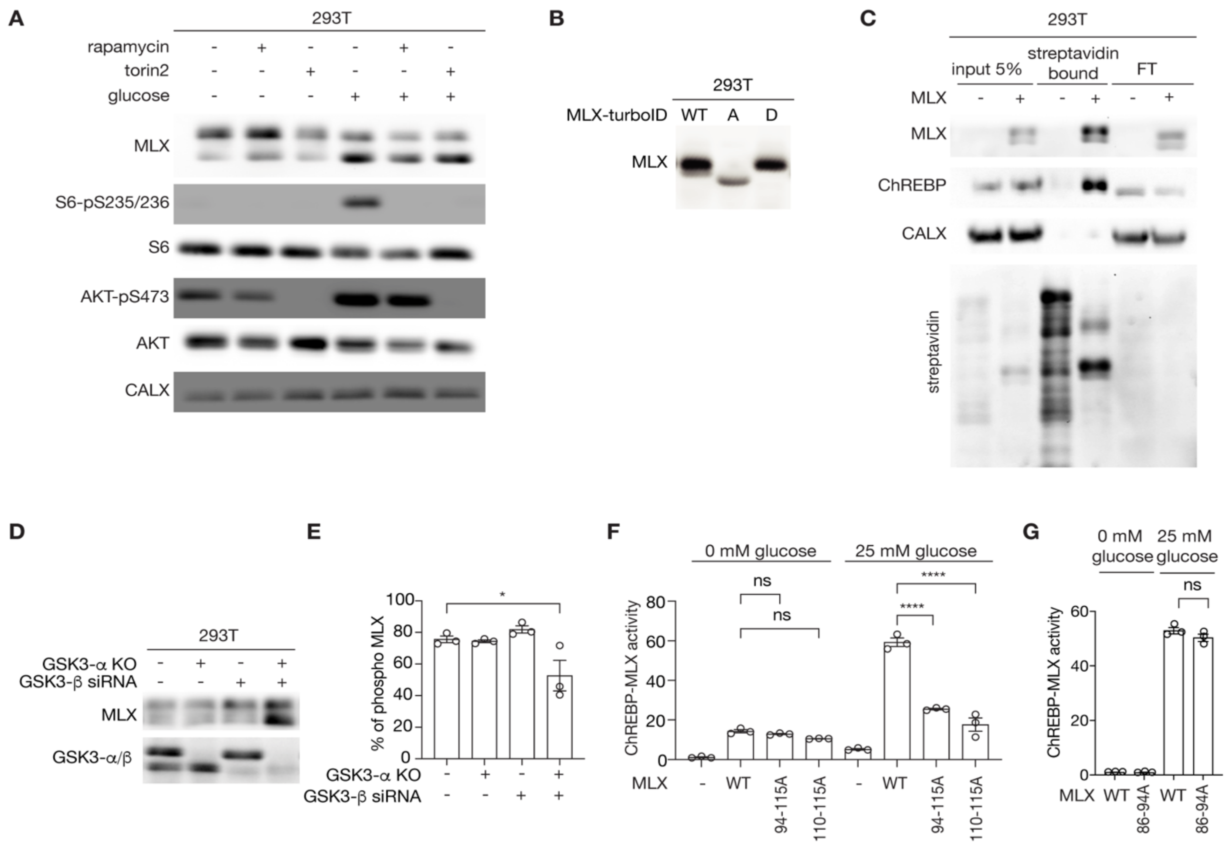

Fig. S4. Identification of CK2 and GSK3 as MLX kinases, related to Fig. 4.

A. MLX phosphorylation in 293T cells treated with the mTORC1 inhibitor rapamycin or the mTORC1/mTORC2 dual inhibitor torin2. Cells were starved for glucose overnight, pretreated with 100 nM rapamycin or 250 nM torin2 for 30 min, and treated with 25 mM glucose for 60 min. S6-pS235/236 and AKT-pS473 serve as positive controls for mTORC1 and mTORC2 inhibition, respectively. CALX serves as a loading control. N=3. **B**. MLX phosphorylation in 293T cells expressing TurboID-tagged MLX- WT, -A and -D. N=3. **C**. Biotinylated proteins in 293T cells expressing TurboID-tagged MLX-WT, HK2 and ChREBP. The streptavidin blot serves as a control for biotinylation. N=3. **D-E**. MLX phosphorylation in WT, GSK3-α KO, GSK3-β KD or GSK3-α/β KO/KD 293T cells. GSK3- α/β serves as a control for KO/KD cells. One-way ANOVA, *p<0.05. N=3. **F**. ChREBP-MLX luciferase reporter activity in 293T cells expressing ChREBP and HK2 with MLX-WT, 94-115A, or 110-115A. Cells were starved for glucose overnight and treated with 25 mM glucose for 3 hours. One-way ANOVA, ****p<0.0001, ns=not significant. N=3. **G.** ChREBP-MLX luciferase reporter activity in 293T cells expressing ChREBP and HK2 with MLX-WT, or 86-94A. Cells were starved for glucose overnight and treated with 25 mM glucose for 3 hours. t-test, ns=not significant. N=3.

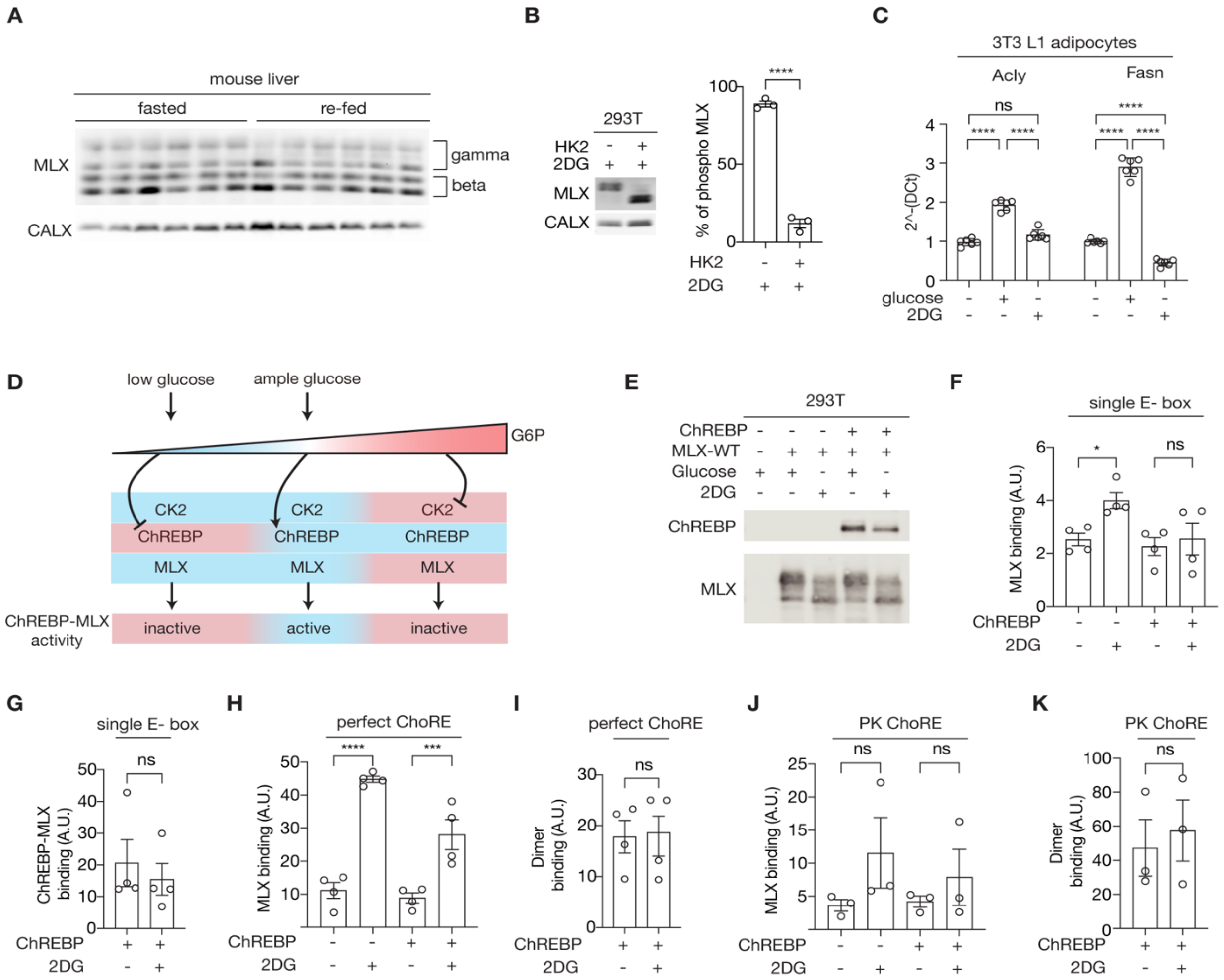

Fig. S5. G6P accumulation inhibits CK2-mediated MLX phosphorylation and the binding of ChREBP-MLX tetramer to the ChoRE, related to Fig. 5.

A. MLX phosphorylation in liver from overnight fasted or overnight fasted and 3-hour re-fed mice. n=6. **B.** MLX phosphorylation in 293T cells transfected with HK2 or empty plasmid. Cells were starved for glucose overnight and re-fed with 2DG for 60 min. CALX serves as a loading control. N=3. **C**. *Acly* and *Fasn* mRNA levels in 3T3 L1 adipocytes treated with 25 mM glucose or 2DG for 30 min. One-way ANOVA, ****p<0.0001, ns=not significant. N=6. **D**. Under basal and starvation conditions, MLX is phosphorylated and primed for activation. Upon glucose uptake, G6P activates ChREBP, facilitating immediate gene expression. As G6P levels increase, CK2-mediated MLX phosphorylation is inhibited, terminating the activity of the ChREBP-MLX complex and thereby halting gene expression. **E**. Immunoprecipitated MLX and ChREBP proteins used for EMSA experiments in Fig. 5J. 293T cells expressing MLX-WT with or without ChREBP were treated with glucose or 2DG for 60 min. **F-K**. Quantification of the data in Fig. 5J. t-test, *p<0.05, ***p<0.001, ****p<0.0001, ns=not significant. N=3-4.

Table S1. Primers used in this study

**Cloning primers**	
Chrebp-HA-fw	CCA GTG TGG TGG AAT TCT GCA TGG CGC GCG CGC TGG CGG ATC TAT CCG TGA AC
Chrebp-HA-rv	GCT GGA TAT CTT TAA GCG TAA TCT GGA ACA TCG TAT GGG TAT AAT GGT CTC
gibson-Mlx-fw	CCA CTA GTC CAG TGT GGT GGG CGA TCG CCA TGA CGG AG
gibson-Mlx-rv	ACT GTG CTG GAT ATC TGC AGT TAA ACC TTA TCG TCG TCA TCC TTG TAA TC
Mlx-alpha/beta-fw	GTC GAG TAT GCC TAC AGT GAC
Mlx-alpha/beta-rv	CTT GAC CCA AGG GTC CTC
Mlx-alpha-fw	GAT GAT GAG GAC AGT GAT TAT CAG
Mlx-alpha-rv	AGG ATC CAG GCT GTT GTC
pUAST-mmMLX-EcoRI-fw	GGG AAT TCC AAA ATG ACG GAG CCG GGC GCC TC
pUAST-mmMLX-XhoI-rv	GGC TCG AGT CAG TAG AGT TGG TTT TTC AAC TGA TGA AGG AC
Mlx-TurboID-fw	GGA CAG CAC CGC TAG CAT GAC GGA GCC GGG CG
Mlx-TurboID-rv	CAC AGT ATT GTC TTT GCT AGC GTA GAG TTG GTT TTT CAA CTG ATG AAG GAC
pGEX-Mlx-fw	GGG AAT TCT TAT GAC GGA GCC GGG CGC CTC
pGEX-Mlx-rv	GGC TCG AGT CAG TAG AGT TGG TTT TTC AAC TGA TGA AGG AC
**Mutagenesis primers**	
Mlx-S91A/S94A-fw	AAG GGG GCT GTA GTG GCC AGA GCT AAT AGC ATC G
Mlx-S86A/T87A-rv	GTG GGC GGC TTC TAC AAA AAG CCC AGG ATC CAG GCT G
Mlx-S101-106A-fw	GCC GCC GCT GCC GCT GCT GTC CCC AAC ACA GAT GAT G
Mlx-S98A-rv	GCC GAT GGC ATT AGC TCT GGA CAC TAC A
Mlx-S115A-fw	GAG GAC GCT GAT TAT CAG CAG GAG TCC TAC AAG
Mlx-T110A-rv	ATC ATC TGC GTT GGG GAC AGA AGA GGC ACT G
Mlx-105-115A-fw	GCT GCT GTC CCC AAC GCA GAT GAT GAG GAC GAT GAT TAT CAG CAG GAG TCC TAC AAG
Mlx-94-103A-rv	GGC AGC GGC GGC GCC GAT GGC ATT AGC TCT GGC CAC TAC ACT CCC CTT GTG GG

Mlx-S101-106D-fw	GAC GAC GAT GCC GAT GAT GTC CCC AAC ACA GAT GAT G
Mlx-S98D-rv	GCC GAT GTC ATT AGC TCT GGA CAC TAC A
Mlx-S115D-fw	GAG GAC GAT GAT TAT CAG CAG GAG TCC TAC AAG
Mlx-T110D-rv	ATC ATC GTC GTT GGG GAC AGA AGA GGC ACT G
Mlx-105-115D-fw	GAT GAT GTC CCC AAC GAC GAT GAT GAG GAC GAT GAT TAT CAG CAG GAG TCC TAC AAG
Mlx-94-103D-rv	GGC ATC GTC GTC GCC GAT GTC ATT AGC TCT GTC CAC TAC ACT CCC CTT GTG GG
Hk2-S155A-fw	TTC ACC TTC GCG TTC CCC TGC CAC CA
Hk2-S155A-rv	CAG GGG AAC GCG AAG GTG AAA CCC AGA GGG
Hk2-S603A-fw	ACA TTC GCC TTC CCT TGC CAG C
Hk2-S603A-rv	AGG GAA GGC GAA TGT GAA ACC CAA AGG C
Chrebp-S140A-fw	CGG AGG AAG GCC CCA GTG TGT GGT TTC G
Chrebp-S140A-rv	CAC TGG GGC CTT CCT CCG TTG CAC ATA CTG
Chrebp-S196A-fw	CGT AAG TCC GCC AGG GAA GGG GAT TTC CTG
Chrebp-S196A-rv	TTC CCT GGC GGA CTT ACG GAG CCG CTT TTT G
Chrebp-S626A-fw	CGG CGA CTA GCC GGG GAT CTC AAC TCC ATA C
Chrebp-S626A-rv	GAG ATC CCC GGC TAG TCG CCG CTC ACT GCC
Chrebp-T665A-fw	CGA CGT ATC GCC CAC ATC TCC GCG GAG CAG
Chrebp-T665A-rv	GGA GAT GTG GGC GAT ACG TCG GTT CTC CAT CTT G
**CRISPR sgRNA and validation primers**	
sgRNA1-Gsk3a fw	CAC CGG GCC ACC CGG TAC ACT GTC T
sgRNA1-Gsk3a-rv	AAA CAG ACA GTG TAC CGG GTG GCC C
sgRNA2-Gsk3a-fw	CAC CGG GAA CTA GTC GCC ATC AAG A
sgRNA2-Gsk3a-rv	AAA CTC TTG ATG GCG ACT AGT TCC C
sgRNA1-Gsk3a-seq-fw	ATG CGT AAG CTG GAC CAC TG
sgRNA1-Gsk3a-seq-rv	ATT CAG TCA GGC CTT GCC TG
sgRNA2-Gsk3a-seq-fw	GTC CCC AAC GAG CTT CCT G
sgRNA2-Gsk3a-seq-rv	CAA GAA TTT GCT TAT TAA GCA CTT ACA TTG
qPCR primers	
mmFasn-fw	GCT GCG GAA ACT TCA GGA AAT
mmFasn-rv	AGA GAC GTG TCA CTC CTG GAC TT
mmAcly-fw	GCC AGC GGG AGC ACA TC
mmAcly-rv	CTT TGC AGG TGC CAC TTC ATC

Table S2. Antibodies used in this study.

**Antibodies**		
anti-ACTIN	Cell Signaling Technology, Inc	Cat#3700, (8H10D10)
anti-AKT (pan)	Cell Signaling Technology, Inc	Cat#2920, (40D4)
anti-Phospho-AKT (Ser473)	Cell Signaling Technology, Inc	Cat#4060, (D9E)
anti-ChREBP	Cell Signaling Technology, Inc	Cat#58069
anti-CK2α	Cell Signaling Technology, Inc	Cat#2656
anti-DYKDDDDK(FLAG) Tag	Cell Signaling Technology, Inc	Cat#8146, (9A3)
anti-GSK3-α/β	Cell Signaling Technology, Inc	Cat#5676, (D75D3)
anti-HA-Tag	Cell Signaling Technology, Inc	Cat#3724, (C29F4)
anti-HA-Tag	Cell Signaling Technology, Inc	Cat#2367, (6E2)
anti-Hexokinase II	Cell Signaling Technology, Inc	Cat#2867, (C64G5)
anti-MLX	Cell Signaling Technology, Inc	Cat#85570, (D8G6W)
anti-S6 Ribosomal Protein	Cell Signaling Technology, Inc	Cat#2217, (5G10)
anti-Phospho-S6 Ribosomal Protein (Ser235/236)	Cell Signaling Technology, Inc	Cat#4856, (2F9)
anti-Calnexin (CALX)	Invitrogen	Cat#PA5-34754
anti-Thiophosphate ester antibody	Abcam	Cat#ab92570
IRDye® 800CW Goat anti-Rabbit IgG	LI-COR Biotech	Cat#926-32211
IRDye® 680RD Goat anti-Mouse IgG Secondary Antibody	LI-COR Biotech	Cat#926-68070
anti-FLAG® M2 Affinity Gel	Sigma-Aldrich	A2220

Table S3. Plasmids used in this study.

**Plasmids**		
pMSCV-*Chrebp(alpha)-FLAG*	Michael Schupp Lab (67)	N/A
pCMV-*Mlx(gamma)-MYC-FLAG*	Origene	Cat#MR204137
pGL3-*ChoRE-Luc*	Michael Schupp Lab (68)	N/A
pNL1.1-*Nluc/TK*	Promega	Cat#N150A
pLenti-Blast-*Gck (isoform2)*	Nissim Hay Lab (69)	N/A
pcDNA3.1-3x*HA-TurboID-NLS*	Alice Ting Lab (29) (Addgene)	Addgene plasmid Cat#107171
lentiCRISPRv2	Feng Zhang Lab (61) (Addgene)	Addgene plasmid Cat#52961
pcDNA3.1-*ChREBP-HA*	This paper	N/A
pcDNA3.1-*Mlx(alpha)-MYC-FLAG*	This paper	N/A
pcDNA3.1-*Mlx(beta)-MYC-FLAG*	This paper	N/A
pcDNA3.1-*Mlx(gamma)-MYC-FLAG*	This paper	N/A
pcDNA3.1-*Mlx(gamma)-A-MYC-FLAG* (S94A, S98A, S101A, T102A, S103A, S105A, S106A, T110A, S115A)	This paper	N/A
pcDNA3.1-*Mlx(gamma)-D-MYC-FLAG* (S94D, S98D, S101D, T102D, S103D, S105D, S106D, T110D, S115D)	This paper	N/A
pcDNA3.1-*Mlx(gamma)-4A-MYC-FLAG* (S86A, T87A, S91A, S94A)	This paper	N/A
pcDNA3.1-*Mlx(gamma)-2-MYC-FLAG A* T110A, S115A	This paper	N/A
pcDNA3.1-*Chrebp(alpha)-A-HA* (S140A, S196A, S626A, T665A)	This paper	N/A
pcDNA3.1-*Hk2*	This paper	N/A
pcDNA3.1-*Hk2-KD* (S155A, S603A)	This paper	N/A
pUAST-*Mlx-WT*	This paper	N/A
pUAST-*Mlx-A*	This paper	N/A
pcDNA3.1-*Mlx-WT-TurboID*	This paper	N/A
pcDNA3.1-*Mlx-A-TurboID*	This paper	N/A
pcDNA3.1-*Mlx-D-TurboID*	This paper	N/A
lentiCRISPRv2-*sgGSK3a*-1	This paper	N/A
lentiCRISPRv2-*sgGSK3a*-2	This paper	N/A
pGEX-*Mlx-WT*	This paper	N/A
pGEX-*Mlx-A*	This paper	N/A

REFERENCES AND NOTES

1. H. Yamashita, M. Takenoshita, M. Sakurai, R. K. Bruick, W. J. Henzel, W. Shillinglaw, D. Arnot, K. Uyeda, A glucose-responsive transcription factor that regulates carbohydrate metabolism in the liver. *Proc. Natl. Acad. Sci. U.S.A.* **98**, 9116–9121 (2001).

2. C. L. Sans, D. J. Satterwhite, C. A. Stoltzman, K. T. Breen, D. E. Ayer, MondoA-Mlx heterodimers are candidate sensors of cellular energy status: Mitochondrial localization and direct regulation of glycolysis. *Mol. Cell. Biol.* **26**, 4863–4871 (2006).

3. S. Ishii, K. Iizuka, B. C. Miller, K. Uyeda, Carbohydrate response element binding protein directly promotes lipogenic enzyme gene transcription. *Proc. Natl. Acad. Sci. U.S.A.* **101**, 15597–15602 (2004).

4. M. V. Li, W. Chen, R. N. Harmancey, A. M. Nuotio-Antar, M. Imamura, P. Saha, H. Taegtmeyer, L. Chan, Glucose-6-phosphate mediates activation of the carbohydrate responsive binding protein (ChREBP). *Biochem. Biophys. Res. Commun.* **395**, 395–400 (2010).

5. R. Dentin, L. Tomas-Cobos, F. Foufelle, J. Leopold, J. Girard, C. Postic, P. Ferré, Glucose 6-phosphate, rather than xylulose 5-phosphate, is required for the activation of ChREBP in response to glucose in the liver. *J. Hepatol.* **56**, 199–209 (2012).

6. M. S. Kim, S. A. Krawczyk, L. Doridot, A. J. Fowler, J. X. Wang, S. A. Trauger, H. L. Noh, H. J. Kang, J. K. Meissen, M. Blatnik, J. K. Kim, M. Lai, M. A. Herman, ChREBP regulates fructose-induced glucose production independently of insulin signaling. *J. Clin. Invest.* **126**, 4372–4386 (2016).

7. A. K. Stoeckman, L. Ma, H. C. Towle, Mlx is the functional heteromeric partner of the carbohydrate response element-binding protein in glucose regulation of lipogenic enzyme genes. *J. Biol. Chem.* **279**, 15662–15669 (2004).

8. K. Iizuka, J. Takeda, Y. Horikawa, Hepatic overexpression of dominant negative Mlx improves metabolic profile in diabetes-prone C57BL/6J mice. *Biochem. Biophys. Res. Commun.* **379**, 499–504 (2009).

9. L. Ma, Y. Y. Sham, K. J. Walters, H. C. Towle, A critical role for the loop region of the basic helix-loop-helix/leucine zipper protein Mlx in DNA binding and glucose-regulated transcription. *Nucleic Acids Res.* **35**, 35–44 (2007).

10. E. Havula, M. Teesalu, T. Hyotylainen, H. Seppala, K. Hasygar, P. Auvinen, M. Oresic, T. Sandmann, V. Hietakangas, Mondo/ChREBP-Mlx-regulated transcriptional network is essential for dietary sugar tolerance in *Drosophila*. *PLOS Genet.* **9**, e1003438 (2013).

11. N. Mejhert, L. Kuruvilla, K. R. Gabriel, S. D. Elliott, M.-A. Guie, H. Wang, Z. W. Lai, E. A. Lane, R. Christiano, N. N. Danial, R. V. Farese Jr., T. C. Walther, Partitioning of MLX-family transcription factors to lipid droplets regulates metabolic gene expression. *Mol. Cell* **77**, 1251–1264.e9 (2020).

12. C. Rufo, M. Teran-Garcia, M. T. Nakamura, S. H. Koo, H. C. Towle, S. D. Clarke, Involvement of a unique carbohydrate-responsive factor in the glucose regulation of rat liver fatty-acid synthase gene transcription. *J. Biol. Chem.* **276**, 21969–21975 (2001).

13. P. Ortega-Prieto, C. Postic, Carbohydrate sensing through the transcription factor ChREBP. *Front. Genet.* **10**, 472 (2019).

14. A. J. Whitmarsh, R. J. Davis, Regulation of transcription factor function by phosphorylation. *Cell. Mol. Life Sci.* **57**, 1172–1183 (2000).

15. G. Meroni, S. Cairo, G. Merla, S. Messali, R. Brent, A. Ballabio, A. Reymond, Mlx, a new Max-like bHLHZip family member: The center stage of a novel transcription factors regulatory pathway? *Oncogene* **19**, 3266–3277 (2000).

16. P. V. Hornbeck, B. Zhang, B. Murray, J. M. Kornhauser, V. Latham, E. Skrzypek, PhosphoSitePlus, 2014: Mutations, PTMs and recalibrations. *Nucleic Acids Res.* **43**, D512–D520 (2015).

17. B. L. O'Callaghan, S. H. Koo, Y. Wu, H. C. Freake, H. C. Towle, Glucose regulation of the acetyl-CoA carboxylase promoter PI in rat hepatocytes. *J. Biol. Chem.* **276**, 16033–16039 (2001).

18. M. Shimobayashi, A. Thomas, S. Shetty, I. C. Frei, B. K. Wolnerhanssen, D. Weissenberger, A. Vandekeere, M. Planque, N. Dietz, D. Ritz, A. C. Meyer-Gerspach, T. Maier, N. Hay, R. Peterli, S. M. Fendt, N. Rohner, M. N. Hall, Diet-induced loss of adipose hexokinase 2 correlates with hyperglycemia. *eLife* **12**, e85103 (2023).

19. T. Kawaguchi, M. Takenoshita, T. Kabashima, K. Uyeda, Glucose and cAMP regulate the L-type pyruvate kinase gene by phosphorylation/dephosphorylation of the carbohydrate response element binding protein. *Proc. Natl. Acad. Sci. U.S.A.* **98**, 13710–13715 (2001).

20. J. Mattila, E. Havula, E. Suominen, M. Teesalu, I. Surakka, R. Hynynen, H. Kilpinen, J. Vaananen, I. Hovatta, R. Kakela, S. Ripatti, T. Sandmann, V. Hietakangas, Mondo-Mlx mediates organismal sugar sensing through the Gli-similar transcription factor sugarbabe. *Cell Rep.* **13**, 350–364 (2015).

21. M. A. Herman, O. D. Peroni, J. Villoria, M. R. Schön, N. A. Abumrad, M. Blüher, S. Klein, B. B. Kahn, A novel ChREBP isoform in adipose tissue regulates systemic glucose metabolism. *Nature* **484**, 333–338 (2012).

22. A. Becker, P. Schlöder, J. E. Steele, G. Wegener, The regulation of trehalose metabolism in insects. *Experientia* **52**, 433–439 (1996).

23. E. Shukla, L. J. Thorat, B. B. Nath, S. M. Gaikwad, Insect trehalase: Physiological significance and potential applications. *Glycobiology* **25**, 357–367 (2015).

24. M. A. Gregory, Y. Qi, S. R. Hann, Phosphorylation by glycogen synthase kinase-3 controls c-myc proteolysis and subnuclear localization. *J. Biol. Chem.* **278**, 51606–51612 (2003).

25. J. Abramson, J. Adler, J. Dunger, R. Evans, T. Green, A. Pritzel, O. Ronneberger, L. Willmore, A. J. Ballard, J. Bambrick, S. W. Bodenstein, D. A. Evans, C.-C. Hung, M. O'Neil, D. Reiman, K. Tunyasuvunakool, Z. Wu, A. Žemgulytė, E. Arvaniti, C. Beattie, O. Bertolli, A. Bridgland, A. Cherepanov, M. Congreve, A. I. Cowen-Rivers, A. Cowie, M. Figurnov, F. B. Fuchs, H. Gladman, R. Jain, Y. A. Khan, C. M. R. Low, K. Perlin, A. Potapenko, P. Savy, S. Singh, A. Stecula, A. Thillaisundaram, C. Tong, S. Yakneen, E. D. Zhong, M. Zielinski, A. Žídek, V.

Bapst, P. Kohli, M. Jaderberg, D. Hassabis, J. M. Jumper, Accurate structure prediction of biomolecular interactions with AlphaFold 3. *Nature* **630**, 493–500 (2024).

26. T. R. Peterson, S. S. Sengupta, T. E. Harris, A. E. Carmack, S. A. Kang, E. Balderas, D. A. Guertin, K. L. Madden, A. E. Carpenter, B. N. Finck, D. M. Sabatini, mTOR complex 1 regulates lipin 1 localization to control the SREBP pathway. *Cell* **146**, 408–420 (2011).

27. A. Hagiwara, M. Cornu, N. Cybulski, P. Polak, C. Betz, F. Trapani, L. Terracciano, M. H. Heim, M. A. Rüegg, M. N. Hall, Hepatic mTORC2 activates glycolysis and lipogenesis through Akt, glucokinase, and SREBP1c. *Cell Metab.* **15**, 725–738 (2012).

28. Y. Tang, M. Wallace, J. Sanchez-Gurmaches, W. Y. Hsiao, H. Li, P. L. Lee, S. Vernia, C. M. Metallo, D. A. Guertin, Adipose tissue mTORC2 regulates ChREBP-driven de novo lipogenesis and hepatic glucose metabolism. *Nat. Commun.* **7**, 11365 (2016).

29. T. C. Branon, J. A. Bosch, A. D. Sanchez, N. D. Udeshi, T. Svinkina, S. A. Carr, J. L. Feldman, N. Perrimon, A. Y. Ting, Efficient proximity labeling in living cells and organisms with TurboID. *Nat. Biotechnol.* **36**, 880–887 (2018).

30. F. Meggio, L. A. Pinna, One-thousand-and-one substrates of protein kinase CK2? *FASEB J.* **17**, 349–368 (2003).

31. F. Pierre, P. C. Chua, S. E. O'Brien, A. Siddiqui-Jain, P. Bourbon, M. Haddach, J. Michaux, J. Nagasawa, M. K. Schwaebe, E. Stefan, A. Vialettes, J. P. Whitten, T. K. Chen, L. Darjania, R. Stansfield, J. Bliesath, D. Drygin, C. Ho, M. Omori, C. Proffitt, N. Streiner, W. G. Rice, D. M. Ryckman, K. Anderes, Pre-clinical characterization of CX-4945, a potent and selective small molecule inhibitor of CK2 for the treatment of cancer. *Mol. Cell. Biochem.* **356**, 37–43 (2011).

32. J. R. Woodgett, P. Cohen, Multisite phosphorylation of glycogen synthase. Molecular basis for the substrate specificity of glycogen synthase kinase-3 and casein kinase-II (glycogen synthase kinase-5). *Biochim. Biophys. Acta* **788**, 339–347 (1984).

33. E. ter Haar, J. T. Coll, D. A. Austen, H. M. Hsiao, L. Swenson, J. Jain, Structure of GSK3beta reveals a primed phosphorylation mechanism. *Nat. Struct. Biol.* **8**, 593–596 (2001).

34. D. B. Ring, K. W. Johnson, E. J. Henriksen, J. M. Nuss, D. Goff, T. R. Kinnick, S. T. Ma, J. W. Reeder, I. Samuels, T. Slabiak, A. S. Wagman, M. E. Hammond, S. D. Harrison, Selective glycogen synthase kinase 3 inhibitors potentiate insulin activation of glucose transport and utilization in vitro and in vivo. *Diabetes* **52**, 588–595 (2003).

35. K. C. Garber, E. E. Carlson, Thiol-ene enabled detection of thiophosphorylated kinase substrates. *ACS Chem. Biol.* **8**, 1671–1676 (2013).

36. S. Jeong, T. T. Nikiforov, Kinase assay based on thiophosphorylation and biotinylation. *Biotechniques* **27**, 1232–1238 (1999).

37. E. van Schaftingen, I. Gerin, The glucose-6-phosphatase system. *Biochem. J.* **362**, 513–532 (2002).

38. A. N. Wick, D. R. Drury, H. I. Nakada, J. B. Wolfe, B. Britton, R. Grabowski, Localization of the primary metabolic block produced by 2-deoxyglucose. *J. Biol. Chem.* **224**, 963–969 (1957).

39. A. Zhu, R. Romero, H. R. Petty, An enzymatic colorimetric assay for glucose-6-phosphate. *Anal. Biochem.* **419**, 266–270 (2011).

40. Z. Liu, S. S. Venkatesh, C. C. Maley, Sequence space coverage, entropy of genomes and the potential to detect non-human DNA in human samples. *BMC Genomics* **9**, 509 (2008).

41. B. Mädge, "E-Box" in *Encyclopedia of Cancer* (2009), chap. 1795, pp. 947–950.

42. X. de Martin, R. Sodaei, G. Santpere, Mechanisms of binding specificity among bHLH transcription factors. *Int. J. Mol. Sci.* **22**, 9150 (2021).

43. C. Grandori, S. M. Cowley, L. P. James, R. N. Eisenman, The Myc/Max/Mad network and the transcriptional control of cell behavior. *Annu. Rev. Cell Dev. Biol.* **16**, 653–699 (2000).

44. R. Gordan, N. Shen, I. Dror, T. Zhou, J. Horton, R. Rohs, M. L. Bulyk, Genomic regions flanking E-box binding sites influence DNA binding specificity of bHLH transcription factors through DNA shape. *Cell Rep.* **3**, 1093–1104 (2013).

45. I. T. Tokuda, A. Okamoto, R. Matsumura, T. Takumi, M. Akashi, Potential contribution of tandem circadian enhancers to nonlinear oscillations in clock gene expression. *Mol. Biol. Cell* **28**, 2333–2342 (2017).

46. A. T. Chang, Y. Liu, K. Ayyanathan, C. Benner, Y. Jiang, J. W. Prokop, H. Paz, D. Wang, H. R. Li, X. D. Fu, F. J. Rauscher 3rd, J. Yang, An evolutionarily conserved DNA architecture determines target specificity of the TWIST family bHLH transcription factors. *Genes Dev.* **29**, 603–616 (2015).

47. F. X. Yu, Y. Luo, Tandem ChoRE and CCAAT motifs and associated factors regulate Txnip expression in response to glucose or adenosine-containing molecules. *PLOS ONE* **4**, e8397 (2009).

48. A. N. Billin, A. L. Eilers, K. L. Coulter, J. S. Logan, D. E. Ayer, MondoA, a novel basic helix-loop-helix-leucine zipper transcriptional activator that constitutes a positive branch of a max-like network. *Mol. Cell. Biol.* **20**, 8845–8854 (2000).

49. A. N. Billin, A. L. Eilers, C. Queva, D. E. Ayer, Mlx, a novel Max-like BHLHZip protein that interacts with the Max network of transcription factors. *J. Biol. Chem.* **274**, 36344–36350 (1999).

50. P. Richards, L. Rachdi, M. Oshima, P. Marchetti, M. Bugliani, M. Armanet, C. Postic, S. Guilmeau, R. Scharfmann, MondoA is an essential glucose-responsive transcription factor in human pancreatic beta-cells. *Diabetes* **67**, 461–472 (2018).

51. F. Rajas, R. Dentin, A. Cannella Miliano, M. Silva, M. Raffin, F. Levavasseur, A. Gautier-Stein, C. Postic, G. Mithieux, The absence of hepatic glucose-6 phosphatase/ChREBP couple is incompatible with survival in mice. *Mol Metab.* **43**, 101108 (2021).

52. R. C. Sun, V. V. Dukhande, Z. Zhou, L. E. A. Young, S. Emanuelle, C. F. Brainson, M. S. Gentry, Nuclear glycogenolysis modulates histone acetylation in human non-small cell lung cancers. *Cell Metab.* **30**, 903–916.e7 (2019).

53. S. E. Roffey, D. W. Litchfield, CK2 Regulation: Perspectives in 2021. *Biomedicine* **9**, 1361 (2021).

54. J. A. Viscarra, Y. Wang, I. H. Hong, H. S. Sul, Transcriptional activation of lipogenesis by insulin requires phosphorylation of MED17 by CK2. *Sci. Signal.* **10**, eaai8596 (2017).

55. G. Di Maira, M. Salvi, G. Arrigoni, O. Marin, S. Sarno, F. Brustolon, L. A. Pinna, M. Ruzzene, Protein kinase CK2 phosphorylates and upregulates Akt/PKB. *Cell Death Differ.* **12**, 668–677 (2005).

56. L. Eissing, T. Scherer, K. Todter, U. Knippschild, J. W. Greve, W. A. Buurman, H. O. Pinnschmidt, S. S. Rensen, A. M. Wolf, A. Bartelt, J. Heeren, C. Buettner, L. Scheja, De novo lipogenesis in human fat and liver is linked to ChREBP-β and metabolic health. *Nat. Commun.* **4**, 1528 (2013).

57. Y. Guri, M. Colombi, E. Dazert, S. K. Hindupur, J. Roszik, S. Moes, P. Jenoe, M. H. Heim, I. Riezman, H. Riezman, M. N. Hall, mTORC2 promotes tumorigenesis via lipid synthesis. *Cancer Cell* **32**, 807–823.e12 (2017).

58. A. Yu, P. Yu, Y. Zhu, R. Zhu, R. Sun, D. Ye, F.-X. Yu, Glucose-induced and ChREBP: MLX-mediated lipogenic program promotes hepatocellular carcinoma development. *Oncogene* **42**, 3182–3193 (2023).

59. P. A. Carroll, D. Diolaiti, L. McFerrin, H. Gu, D. Djukovic, J. Du, P. F. Cheng, S. Anderson, M. Ulrich, J. B. Hurley, D. Raftery, D. E. Ayer, R. N. Eisenman, Deregulated Myc requires MondoA/Mlx for metabolic reprogramming and tumorigenesis. *Cancer Cell* **27**, 271–285 (2015).

60. H. Wang, J. Lu, F. Alencastro, A. Roberts, J. Fiedor, P. Carroll, R. N. Eisenman, S. Ranganathan, M. Torbenson, A. W. Duncan, E. V. Prochownik, Coordinated cross-talk between the Myc and Mlx networks in liver regeneration and neoplasia. *Cell. Mol. Gastroenterol. Hepatol.* **13**, 1785–1804 (2022).

61. N. E. Sanjana, O. Shalem, F. Zhang, Improved vectors and genome-wide libraries for CRISPR screening. *Nat. Methods* **11**, 783–784 (2014).

62. W. Zhang, B. J. Thompson, V. Hietakangas, S. M. Cohen, MAPK/ERK signaling regulates insulin sensitivity to control glucose metabolism in Drosophila. *PLOS Genet.* **7**, e1002429 (2011).

63. J. M. Tennessen, W. E. Barry, J. Cox, C. S. Thummel, Methods for studying metabolism in Drosophila. *Methods* **68**, 105–115 (2014).

64. E. F. Pettersen, T. D. Goddard, C. C. Huang, E. C. Meng, G. S. Couch, T. I. Croll, J. H. Morris, T. E. Ferrin, UCSF ChimeraX: Structure visualization for researchers, educators, and developers. *Protein Sci.* **30**, 70–82 (2021).

65. F. Madeira, M. Pearce, A. R. N. Tivey, P. Basutkar, J. Lee, O. Edbali, N. Madhusoodanan, A. Kolesnikov, R. Lopez, Search and sequence analysis tools services from EMBL-EBI in 2022. *Nucleic Acids Res.* **50**, W276–W279 (2022).

66. Y. Perez-Riverol, J. Bai, C. Bandla, D. Garcia-Seisdedos, S. Hewapathirana, S. Kamatchinathan, D. J. Kundu, A. Prakash, A. Frericks-Zipper, M. Eisenacher, M. Walzer, S. Wang, A. Brazma, J. A. Vizcaino, The PRIDE database resources in 2022: A hub for mass spectrometry-based proteomics evidences. *Nucleic Acids Res.* **50**, D543–D552 (2022).

67. N. Witte, M. Muenzner, J. Rietscher, M. Knauer, S. Heidenreich, A. M. Nuotio-Antar, F. A. Graef, R. Fedders, A. Tolkachov, I. Goehring, M. Schupp, The glucose sensor ChREBP links de novo lipogenesis to PPARgamma activity and adipocyte differentiation. *Endocrinology* **156**, 4008–4019 (2015).

68. S. Heidenreich, P. Weber, H. Stephanowitz, K. M. Petricek, T. Schutte, M. Oster, A. M. Salo, M. Knauer, I. Goehring, N. Yang, N. Witte, A. Schumann, M. Sommerfeld, M. Muenzner, J. Myllyharju, E. Krause, M. Schupp, The glucose-sensing transcription factor ChREBP is targeted by proline hydroxylation. *J. Biol. Chem.* **295**, 17158–17168 (2020).

69. D. DeWaal, V. Nogueira, A. R. Terry, K. C. Patra, S. M. Jeon, G. Guzman, J. Au, C. P. Long, M. R. Antoniewicz, N. Hay, Hexokinase-2 depletion inhibits glycolysis and induces oxidative

phosphorylation in hepatocellular carcinoma and sensitizes to metformin. *Nat. Commun.* **9**, 446 (2018).
